# Glycolytic-to-oxidative fiber-type switch and mTOR signaling activation are early-onset features of SBMA muscle modified by high-fat diet

**DOI:** 10.1007/s00401-016-1550-4

**Published:** 2016-03-12

**Authors:** Anna Rocchi, Carmelo Milioto, Sara Parodi, Andrea Armirotti, Doriana Borgia, Matteo Pellegrini, Anna Urciuolo, Sibilla Molon, Valeria Morbidoni, Manuela Marabita, Vanina Romanello, Pamela Gatto, Bert Blaauw, Paolo Bonaldo, Fabio Sambataro, Diane M. Robins, Andrew P. Lieberman, Gianni Sorarù, Lodovica Vergani, Marco Sandri, Maria Pennuto

**Affiliations:** Department of Neuroscience and Brain Technologies, Istituto Italiano di Tecnologia, 16163 Genoa, Italy; Dulbecco Telethon Institute, Centre for Integrative Biology, University of Trento, 38123 Trento, Italy; Dipartimento di Medicina Sperimentale, University of Genova, 16100 Genoa, Italy; Neurogenetics Branch, NINDS, National Institutes of Health, Bethesda, MD 20892 USA; Drug Discovery and Development Department, Istituto Italiano di Tecnologia, 16100 Genoa, Italy; Department of Neurosciences, University of Padova, 35100 Padua, Italy; Department of Molecular Medicine, University of Padova, 35131 Padua, Italy; Venetian Institute of Molecular Medicine, Department of Biomedical Science, University of Padova, 35100 Padua, Italy; High-Throughput Screening Core Facility, Centre for Integrative Biology, University of Trento, 38123 Trento, Italy; Department of Experimental and Clinical Medical Sciences (DISM), University of Udine, 33100 Udine, Italy; Department of Human Genetics, University of Michigan Medical School, Ann Arbor, MI 48109 USA; Department of Pathology, University of Michigan Medical School, Ann Arbor, MI 48109 USA; Telethon Institute of Genetic and Medicine, Pozzuoli, 80100 Naples, Italy; Department of Experimental Medicine (DIMES)-Biochemistry Section, University of Genova, Viale Benedetto XV, 1, 16132 Genoa, Italy

**Keywords:** Spinal and bulbar muscular atrophy, Androgen receptor, Skeletal muscle, mTOR, Rapamycin, PGC1α, High-fat diet

## Abstract

Spinal and bulbar muscular atrophy (SBMA) is a neuromuscular disease caused by the expansion of a polyglutamine tract in the androgen receptor (AR). The mechanism by which expansion of polyglutamine in AR causes muscle atrophy is unknown. Here, we investigated pathological pathways underlying muscle atrophy in SBMA knock-in mice and patients. We show that glycolytic muscles were more severely affected than oxidative muscles in SBMA knock-in mice. Muscle atrophy was associated with early-onset, progressive glycolytic-to-oxidative fiber-type switch. Whole genome microarray and untargeted lipidomic analyses revealed enhanced lipid metabolism and impaired glycolysis selectively in muscle. These metabolic changes occurred before denervation and were associated with a concurrent enhancement of mechanistic target of rapamycin (mTOR) signaling, which induced peroxisome proliferator-activated receptor γ coactivator 1 alpha (PGC1α) expression. At later stages of disease, we detected mitochondrial membrane depolarization, enhanced transcription factor EB (TFEB) expression and autophagy, and mTOR-induced protein synthesis. Several of these abnormalities were detected in the muscle of SBMA patients. Feeding knock-in mice a high-fat diet (HFD) restored mTOR activation, decreased the expression of PGC1α, TFEB, and genes involved in oxidative metabolism, reduced mitochondrial abnormalities, ameliorated muscle pathology, and extended survival. These findings show early-onset and intrinsic metabolic alterations in SBMA muscle and link lipid/glucose metabolism to pathogenesis. Moreover, our results highlight an HFD regime as a promising approach to support SBMA patients.

**Electronic supplementary material:**

The online version of this article (doi:10.1007/s00401-016-1550-4) contains supplementary material, which is available to authorized users.

## Introduction

Spinal and bulbar muscular atrophy (SBMA) is an X-linked motor neuron disease characterized by late-onset degeneration of lower motor neurons and skeletal muscle atrophy [41]. In addition, SBMA patients can develop non-motor symptoms, such as sexual dysfunctions, diabetes, and severe urinary tract dysfunction, which are related to androgen insensitivity [75]. SBMA is caused by the expansion of a polyglutamine tract in the gene encoding AR [43]. Polyglutamine expansions in specific genes are responsible for eight other neurodegenerative diseases, namely Huntington’s disease (HD), dentatorubral–pallidoluysian atrophy, and spinocerebellar ataxia (SCA) type 1, 2, 3, 6, 7, and 17 [67]. A unique feature of SBMA in the family of polyglutamine diseases is sex specificity: full manifestations are restricted to males. Sex specificity is due to the conversion of polyglutamine-expanded AR to a toxic species that occurs upon binding to its natural ligand, testosterone. In its inactive state, AR localizes to cytosol. Binding of AR to testosterone results in nuclear translocation, DNA binding, interaction with transcriptional co-regulators, and regulation (activation and repression) of expression of androgen-responsive genes; these post-translational events have all been associated with pathogenesis [71]. Although several experimental and clinical strategies to modify disease have been tested to date [81], no effective therapy for SBMA has yet been developed.

Skeletal muscle (dys)function is emerging as a key component of SBMA pathogenesis [84]. In addition to selective degeneration of motor neurons, SBMA patients develop progressive skeletal muscle weakness, fasciculations, and atrophy. SBMA patients show signs of muscle denervation, such as nerve sprouting, muscle fiber atrophy, and fiber-type grouping, together with signs of muscle degeneration, such as splitting, presence of central nuclei, and degeneration of fibers [96]. Neurogenic and myopathic processes have both been described in mouse models of SBMA [72, 84]. Notably, knock-in SBMA mice show muscle pathology that precedes spinal cord pathology [106]. Consistent with the idea that muscle plays a key role in pathogenesis, targeted overexpression of insulin-like growth factor 1 (IGF-1) selectively in muscle ameliorated disease manifestations in transgenic mice [70]. Unequivocal genetic evidence for a primary role of muscle in SBMA pathogenesis was recently provided by the observation that expression of polyglutamine-expanded AR in all tissues except skeletal muscle was sufficient to prevent disease manifestations in transgenic mice [17]. Supporting these observations were concurrent studies demonstrating that knockdown of polyglutamine-expanded AR expression only in peripheral tissues by antisense oligonucleotides was sufficient to attenuate disease manifestations in SBMA knock-in mice [45]. However, how polyglutamine-expanded AR causes muscle atrophy remains to be clarified.

Alterations of metabolic pathways and of the nutrient and energy status at the organism and cell levels are emerging as key players in neurodegenerative diseases [11]. Skeletal muscle is a central nutrient- and stress-sensor and has a primary role in age-related neurodegenerative diseases [24]. Skeletal muscle regulates systemic aging by influencing metabolism and responses of the organism to dietary restriction and oxidative stress; as such, it affects both life span and the progression of age-related diseases [3, 25]. The homeostasis of skeletal muscle is maintained by a fine balance between anabolic and catabolic processes, which co-exist in a dynamic equilibrium to regulate the rate of protein synthesis and degradation (i.e., protein turnover) as well as muscle fiber size. Muscle hypertrophy is characterized by an increase in muscle mass, which results from increased protein synthesis rate and muscle fiber size. On the other hand, muscle atrophy is characterized by reduced muscle mass, which results from reduced fiber size, increased protein degradation, and decreased force production and fatigue resistance. This equilibrium is maintained by several signaling pathways, including the evolutionarily conserved IGF-1/Akt pathway [91]. Based on the central role of skeletal muscle in energy metabolism and the fact that muscle is an easier therapeutic target compared to the nervous system for neurodegenerative diseases, here we investigated pathological processes occurring in SBMA muscles.

## Materials and methods

### Animals and treatments

Animal care and experimental procedures were conducted in accordance with the Italian Institute of Technology and the University of Trento ethics committees and were approved by the Italian Ministry of Health. Generation and genotyping of knock-in AR21Q and AR113Q mice were previously described [106]. Mice were randomized and fed a standard diet (Mucedola 4RF21), purified normal chow diet (NCD, D12450B Research Diet), and high-fat diet (HFD, D12451 Research Diet) as indicated. The operator was blind for genotype and treatment. For rotarod analysis (Ugo Basile), mice received a weekly session, which included one trial followed by two test trials at 21 rpm speed for a maximum period of 600 s and the average of recordings was used. For rapamycin treatment, 154-day-old mice were injected intraperitoneally with rapamycin (4.0 mg/kg, Gold Biotechnology). For analysis of the rate of protein synthesis, animals were starved 30 min, injected with puromycin (0.040 μmol/g puromycin dissolved in 100 μl PBS), and sacrificed 30 min after injection. In vivo and ex vivo muscle force was measured as previously described [8].

### Human samples

Anonymized control and patient biopsy sample collection was approved by the ethics committee of the University of Padova (Italy). Written informed consent was obtained from each patient. All patients who underwent muscle biopsy were clinically affected and showed weakness and/or fasciculation and/or muscle atrophy. Myopathic changes together with neurogenic atrophy were observed in muscle biopsies.

### Histological analysis

Muscles collected immediately after euthanasia were flash-frozen in isopentane precooled in liquid nitrogen and embedded in optimal cutting temperature (OCT) compound (Tissue Tek, Sakura), and cross sections (10 µm thick) were cut with a cryostat (CM1850 UV, Leica Microsystems). Cryosections were processed for hematoxylin and eosin (H/E) and nicotinamide adenine dinucleotide (NADH) staining, as previously described [70]. For immunofluorescence analysis, muscle cryosections were incubated with M.O.M. IgG blocking solution (Vector), washed with phosphate-buffered saline (PBS) and incubated with a solution of PBS containing 0.5 % bovine serum albumin (BSA). Full list of antibodies used is provided in the Supplementary Information. Sections were mounted with aqueous mounting medium (Fluorescence Mounting Medium, Dako). Images were taken using an upright epifluorescence microscope (Zeiss Axio Imager M2) equipped with an X-Cite 120Q fluorescence light source and a Zeiss Mrm Color Camera. Multichannel images and mosaics were taken using Zeiss Axio Vision Software (V.4.8.2 SP3).

### Biochemical analysis

Muscles were mechanically pulverized and homogenized in lysis buffer [20 mM HEPES, 5 mM EGTA, 2 % sodium dodecyl sulfate (SDS)] and processed by SDS–polyacrylamide gels (SDS-PAGE) as previously described [70]. Full list of antibodies used is provided in the Supplementary Information. Signal intensities were quantified by ImageQuant LAS 4000 mini (GE Healthcare BioSciences). Lactate dehydrogenase (LDH) and citrate synthase activity was measured as previously described [42, 99]. For proteasome activity assay, muscles were lysed using ice-cold lysis buffer [50 mM HEPES, 5 mM EDTA, 150 mM NaCl, 2 mM ATP, 1 % Triton X-100], sonicated and centrifuged. Protein extracts were incubated in 25 mM HEPES, 0.5 mM EDTA, 0.05 % NP40, 0.001 % SDS, and 0.5 mM *N*-Succinyl-Leu-Leu-Val-Tyr-7-Amido-4-Methylcoumarin (Sigma). The absorption was recorded at 460 nm by excitation at 355 nm (Victor3-V luminometer, PerkinElmer).

### High-resolution LC–MS/MS analysis for lipidomic profiling, real-time PCR, and microarray hybridization and data acquisition

Untargeted metabolomics experiments were performed as previously described [2]. For real-time analysis, total RNA was extracted with Trizol (Invitrogen), purified using RNeasy MinElute Cleanup Kit (QIAGEN), and reverse transcribed into cDNA using the iScript Reverse Transcription Supermix (Bio-Rad). Gene expression was measured by quantitative real-time PCR using 7900 HT Fast Real-Time PCR System (Applied Biosystems). The list of primers is provided in Supplementary Table 1. For microarray analysis, total RNA was purified using RNeasy MinElute Cleanup Kit (QIAGEN). High-quality RNA was used for labeling and array hybridization (Agilent Mouse GE 4X44 K slides). Slides were scanned on the Agilent DNA Microarray Scanner (G2505C) using the AgilentHD_GX_1Color Profile (scan area: 61 × 21.6 mm; scan resolution: 5 μm, dye channel set to 100 % Green PMT) of Agilent ScanControl software 8.1.3. Images were analyzed with Feature Extraction Software 10.7.3.1 (Agilent Technologies) using default parameters (protocol GE1_107_Sep09). Expression data were analyzed using the limma package from *R* and false discovery rate (FDR) control for statistical assessment of the microarray data (corrected *P* < 0.05 were considered significant). Gene set enrichment analysis (GSEA) was performed to identify sets of related genes altered in each experimental group (http://www.broad.mit.edu/gsea). Heat maps were generated using MeV TM4 software V 4.9 (http://www.tm4.org). Microarray data are available at the Gene Expression Omnibus database under accession number GSE68441.

### Mitochondrial membrane potential and complex activity analyses

Mitochondrial membrane potential was measured in isolated fibers from flexor digitorum brevis muscles. Mitochondrial membrane potential was measured by epifluorescence microscopy based on the accumulation of tetramethyl rhodamine methyl ester (TMRM) fluorescence, as previously described [82]. For mitochondrial complex activity, total muscle lysates were extracted and the enzymatic activities of the respiratory chain complexes I–IV were assayed as previously described [99].

### Statistical analysis

All data are presented as mean ± standard error of the mean (SEM). Statistical differences of continuous data from two experimental groups were calculated using two-sample *t* tests. Comparisons of data from more than two groups were performed using a one-way ANOVA followed by a Fisher’s least significant difference post hoc test. Statistical comparisons of lifespan curves were performed using the log-rank and Gehan–Breslow–Wilcoxon tests. Statistical significance threshold was set at *P* < 0.05, unless otherwise indicated.

## Results

### Shift towards oxidative metabolism in glycolytic SBMA muscles

**Fig. 1:**
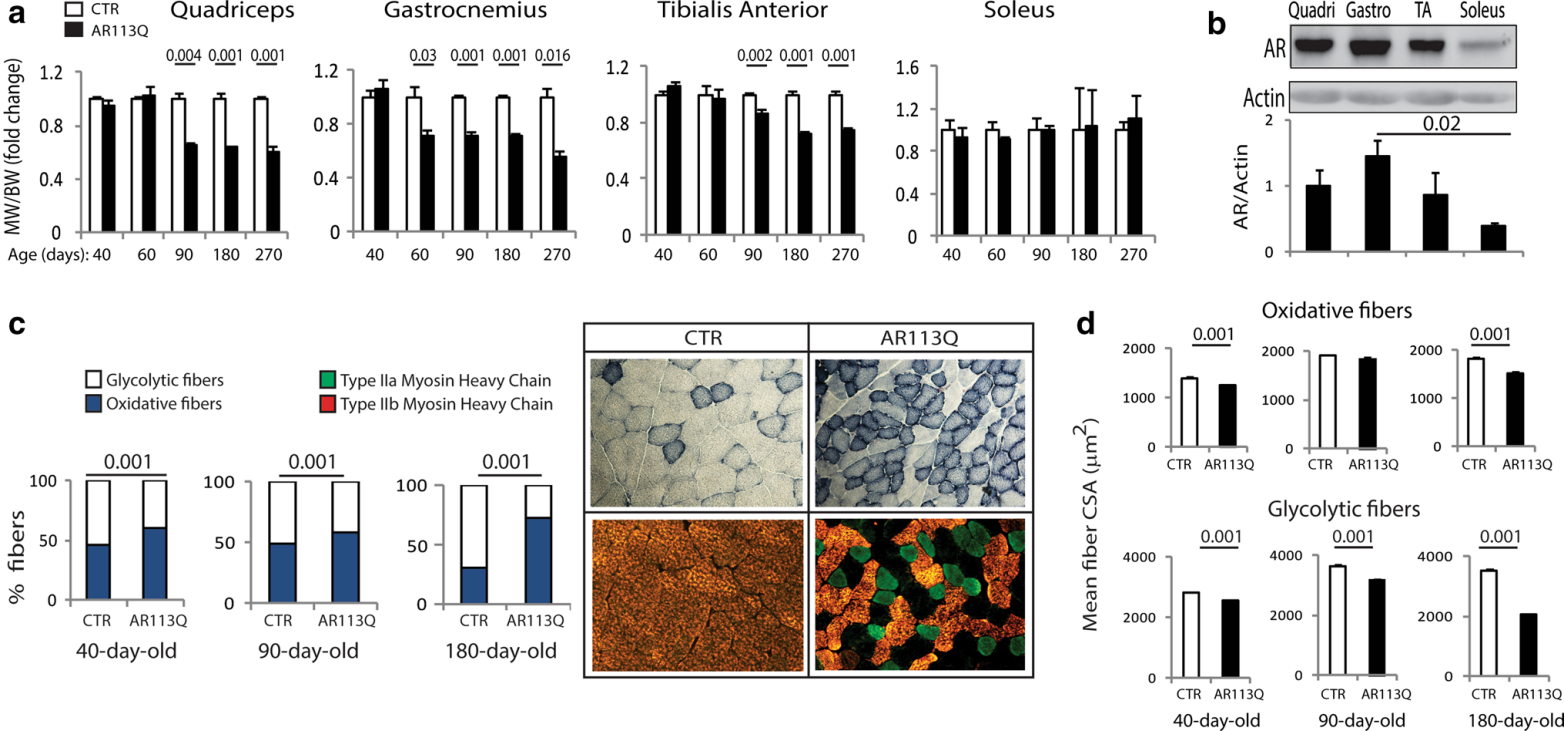
Glycolytic-to-oxidative fiber-type switch in SBMA glycolytic muscles. **a** Muscle weight (MW) normalized to body weight (BW) of the indicated skeletal muscles of AR113Q and control (CTR, wild type) mice analyzed as a function of disease progression. Graphs show mean ± sem, *n* = 3–10 mice. **b** Western blotting analysis of AR expression levels in quadriceps (quadri), gastrocnemius (gastro), tibialis anterior (TA), and soleus muscles of 90-day-old AR113Q mice. AR was detected with specific antibody, and actin was used as loading control. Graph shows mean ± sem, *n* = 3. **c** *Left* NADH staining of quadriceps from AR113Q and CTR (wild type) mice. Graphs show mean ± sem, *n* = 3–8 mice. *Right* NADH staining (*top panel*) and immunofluorescence of type IIa (*green*) and IIb (*red*) myosin heavy chain-positive fibers (*bottom panel*) in the quadriceps muscle of 180-day-old AR113Q and CTR mice. Shown are representative images from *n* = 3 mice. **d** Analysis of the mean oxidative and glycolytic myofiber CSA in the quadriceps of AR113Q and CTR (wild type) mice. Graphs show mean ± sem, *n* = 3 mice

To elucidate the pathological processes underlying muscle atrophy in SBMA, we used SBMA knock-in mice that express AR with a pathogenic polyglutamine tract of 113 glutamine residues (AR113Q) and, as controls, AR21Q and wild-type littermates [72]. No signs of muscle atrophy were detected in AR21Q mice (Supplementary Fig. 1a), as previously described [107]. To determine whether expression of polyglutamine-expanded AR differentially affects muscles composed primarily of either type I slow-oxidative fibers (e.g., soleus) or type II fast-glycolytic fibers [e.g., quadriceps, gastrocnemius, tibialis anterior (TA)], we assessed the wet weight of these muscles as a function of disease progression. The mass of quadriceps, gastrocnemius, and TA was reduced by 13–35 % beginning at 60 or 90 days of age, whereas the mass of soleus did not change over the course of disease in AR113Q mice compared to age-matched control mice (Fig. 1a; Supplementary Fig. 1b). By H/E analysis, the mean myofiber cross-sectional area (CSA) of quadriceps, gastrocnemius, and TA muscles was decreased by 10–30 % in 90- and 180-day-old AR113Q mice, whereas that of soleus was increased (Supplementary Fig. 2a–c). Tetanic force production was decreased by 27 % in the gastrocnemius, but not soleus (Supplementary Fig. 1c, d), indicating that structural alterations are associated with functional deficits. Notably, the degree of atrophy correlated with the levels of expression of AR, suggesting a dose-dependent effect of polyglutamine-expanded AR in muscle (Fig. 1b). These results indicate that expression of AR113Q preferentially affects glycolytic muscles compared to oxidative muscles.

Analysis of muscle biopsies has revealed that the number of type I oxidative fibers is increased in the glycolytic muscles of SBMA patients, suggesting metabolic alterations [38]. By NADH staining, we observed a progressive shift from fast-glycolytic towards slow-oxidative fiber subtype in the quadriceps and gastrocnemius of AR113Q mice, which was detected as early as 40 days of age (Fig. 1c; Supplementary Fig. 3a, b). Staining of type IIa (fast-oxidative) and IIb (fast-glycolytic) fibers confirmed that the number of type IIb fibers was decreased, whereas that of type IIa was increased in the quadriceps of AR113Q mice. Notably, the mean CSA of oxidative fibers was decreased by 10 % in 40-day-old AR113Q mice and progressed to 16 % in 180-day-old mice, whereas that of glycolytic fibers was decreased by 10 % at 40 days of age and progressed to 40 % at 180 days of age in the quadriceps and gastrocnemius (Fig. 1d; Supplementary Fig. 4a, b). These results indicate an early-onset glycolytic-to-oxidative fiber-type switch with atrophy of glycolytic fibers exceeding that of oxidative fibers in the muscle of SBMA knock-in mice.

### Glycolysis is impaired in SBMA muscles

**Fig. 2:**
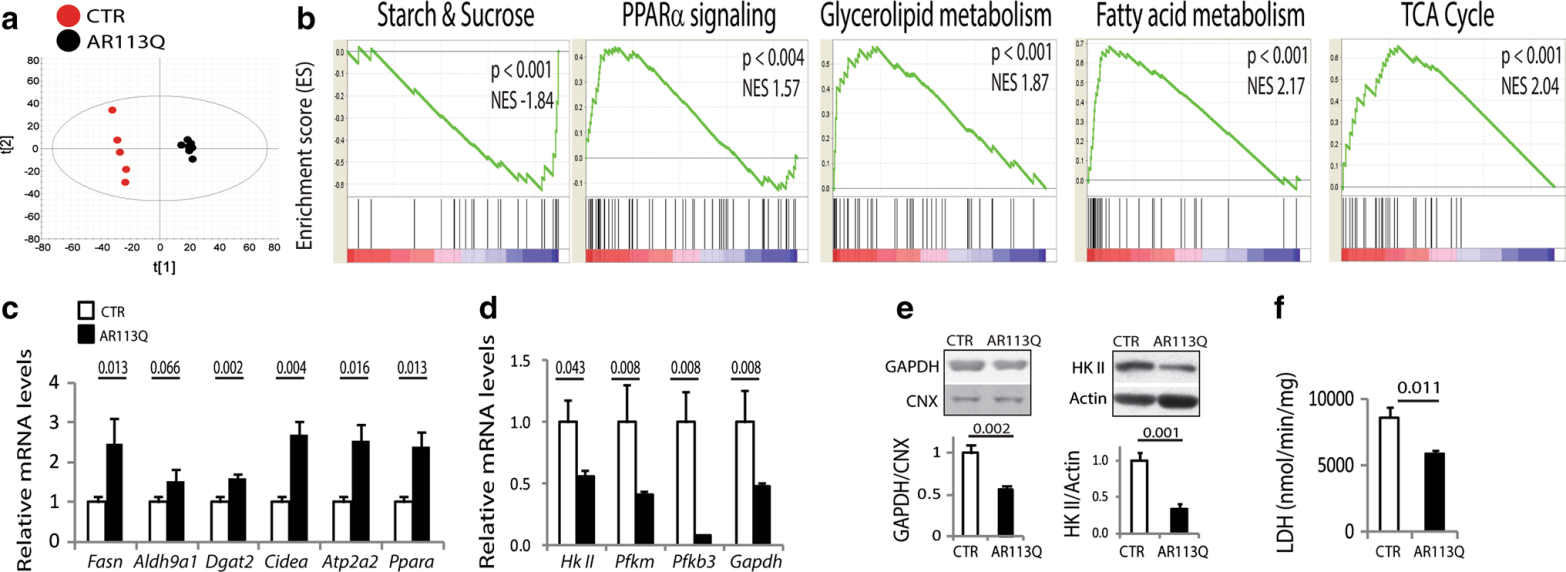
Lipid metabolism is enhanced and glycolysis is impaired in the glycolytic muscles of SBMA knock-in mice. **a** Score plot from principal component analysis of high-resolution LC–MS/MS shotgun lipidomic analysis in the quadriceps muscle of 180-day-old AR113Q and CTR (wild type) mice (*n* = 6–7 mice). **b** GSEA analysis of pathways enriched in genes differentially expressed in AR113Q mice compared to age-matched CTR (wild type) mice. *NES* normalized enrichment score. **c**, **d** Real-time PCR analysis of the transcript levels of lipid and glycolytic genes normalized to beta-glucuronidase in the quadriceps muscle of 180-day-old AR113Q and CTR (wild type) mice. Graph shows mean ± sem, *n* = 5 mice. **e** Western blotting analysis of glycolytic protein expression levels in the muscle of 180-day-old AR113Q and CTR (wild type) mice. Graphs show mean ± sem, *n* = 5 mice. Calnexin (CNX) and actin were used as loading control. **f** Enzymatic activity of lactate dehydrogenase (LDH) in the quadriceps of 180-day-old AR113Q and CTR (wild type) mice. Graph shows mean ± sem, *n* = 3–5 mice

Given that oxidative and glycolytic fibers primarily use oxidative phosphorylation and glycolysis to generate ATP, respectively, we hypothesized that the lipid profile of AR113Q mice was altered. Using untargeted high-resolution LC–MS/MS lipidomic and unsupervised principal component analyses, we identified major lipid alterations in muscle, but not serum, with 45 lipids significantly (*P* ≤ 0.001) enriched (fold change >1.5) and 22 lipids decreased (fold change <0.5) in AR113Q mice (Fig. 2a; Supplementary Fig. 5a; Supplementary Table 2). Lysophosphatidylcholines, lysophosphatidylethanolamines, ceramides, diglycerides, and polyunsaturated fatty acids were increased, whereas species enriched in polyunsaturated lipids and phosphatidylglycerol were decreased, suggesting increased lipid synthesis and turnover. To investigate the molecular pathways underlying the metabolic alterations detected in glycolytic SBMA muscles, we performed whole genome microarray analysis on quadriceps muscle. We identified 550 upregulated (fold change ≥1.5) genes and 286 downregulated (fold change ≤0.6) genes in the quadriceps of 180-day-old AR113Q mice compared to age-matched control mice (*P* < 0.05, false discovery rate <0.05) (Supplementary Fig. 5b; Supplementary Table 3). Consistent with the glycolytic-to-oxidative metabolic shift described above, the transcript levels of markers of oxidative fibers, such as troponin I1 (*Tnni1*), troponin C1 (*Tnnc1*), troponin T1 (*Tnnt1*), ankyrin repeat domain 2 (*Ankrd2*), myoglobin (*Mb*), myosin heavy chain 7b (*Myh7b*), were all upregulated, whereas those of glycolytic fibers were either decreased, such as 5′-AMP-activated protein kinase subunit gamma-3 (*Prkag3*), or remained unchanged, such as troponin I2 (*TnnI2*), myosin heavy chain 4 (*Myh4*), and parvalbumin (*Pvalb*) (Supplementary Table 4). Gene set enrichment analysis (GSEA) revealed that the top pathways enriched in genes differentially expressed in AR113Q and control mice were metabolic pathways (Fig. 2b; Supplementary Table 5). The gene set “starch/sucrose metabolism”, which includes glycolytic genes, was downregulated, whereas “peroxisome proliferator-activated receptor alpha (PPARα) signaling”, “glycerolipid and fatty acid metabolism”, and “tricarboxylic acid (TCA) cycle” were upregulated. Similar changes in metabolic pathways were also detected in transgenic mice overexpressing AR97Q (Supplementary Table 5) [57]. By real-time PCR analysis, we validated the upregulation of genes involved in lipid metabolism, such as fatty acid synthase (*Fasn*), which catalyzes the synthesis of palmitate from malonyl-coenzyme A and acetyl-coenzyme A in a process that requires NADPH, aldehyde dehydrogenase 9 subfamily A1 (*Aldh9a1*), which detoxifies from accumulation of aldehydes generated by lipid peroxidation through NAD(P)+-dependent oxidation to carboxylic acids, diacylglycerol O-acyltransferase 2 (*Dgat2*), which catalyzes the synthesis of triglycerides from diacylglycerol and acyl-coenzyme A, the mitochondrial protein cell death-inducing DNA fragmentation factor alpha subunit-like effector A (*Cidea*), which plays a role in thermogenesis and lipolysis, the sarcoplasmic reticulum SERCA Ca(2+)-ATPase A2 (*Atp2a2*), and *Ppara*, which is involved in the regulation of muscle metabolism (Fig. 2c). Importantly, the expression of these genes was not altered in soleus (Supplementary Fig. 6a). The expression and activity of key enzymes in the glycolytic pathway, such as hexokinase II (*Hk II*), phosphofructokinase 1 (*Pfkm*), 6-phosphofructo-2-kinase/fructose-2,6-biphosphatase 3 (*Pfkb3*), glyceraldehyde 3-phosphate dehydrogenase (*Gapdh*), phosphoglycerate kinase 1 (*Pgk1*), and lactate dehydrogenase (LDH), were downregulated in the quadriceps muscle of AR113Q mice, whereas the activity of citrate synthase (CS) was increased (Fig. 2d–f; Supplementary Fig. 5c, d). Alterations in glycolytic gene expression preceded those in lipid gene expression, as they were detected as early as 90 days of age in the quadriceps of AR113Q mice (Supplementary Fig. 7a, b). In soleus muscle, the transcript levels of *Gapdh*, and not the other glycolytic genes, were decreased by 40 % at 180 days of age (Supplementary Fig. 6b). Our microarray analysis also revealed that the transcript levels of *SLC2A3*, which encodes the glucose transporter 3 (GLUT3), were decreased by 60 %, suggesting altered glucose uptake, whereas those of *SLC2A5*, which encodes the solute carrier family 2 (facilitated glucose/fructose transporter), member 5 (GLUT5), was upregulated by 4.7-fold in the muscle of AR113Q mice, suggesting compensatory mechanisms for carbohydrate uptake (Supplementary Table 2). Fasting serum glucose and insulin levels were similar in AR113Q and control mice (Supplementary Fig. 8a), and intraperitoneal glucose and insulin tolerance tests showed normal glucose clearance and insulin sensitivity in AR113Q mice (Supplementary Fig. 8b, c). Moreover, no overt alterations in glycogen storage were detected in the quadriceps of 180-day-old AR113Q mice and SBMA patients (Supplementary Fig. 9). Taken together, these results indicate that glycolytic muscles in SBMA knock-in mice show enhanced lipid metabolism and gene expression programs, as well as impaired glycolysis.

### Unbalanced protein synthesis and degradation in glycolytic SBMA muscles

**Fig. 3:**
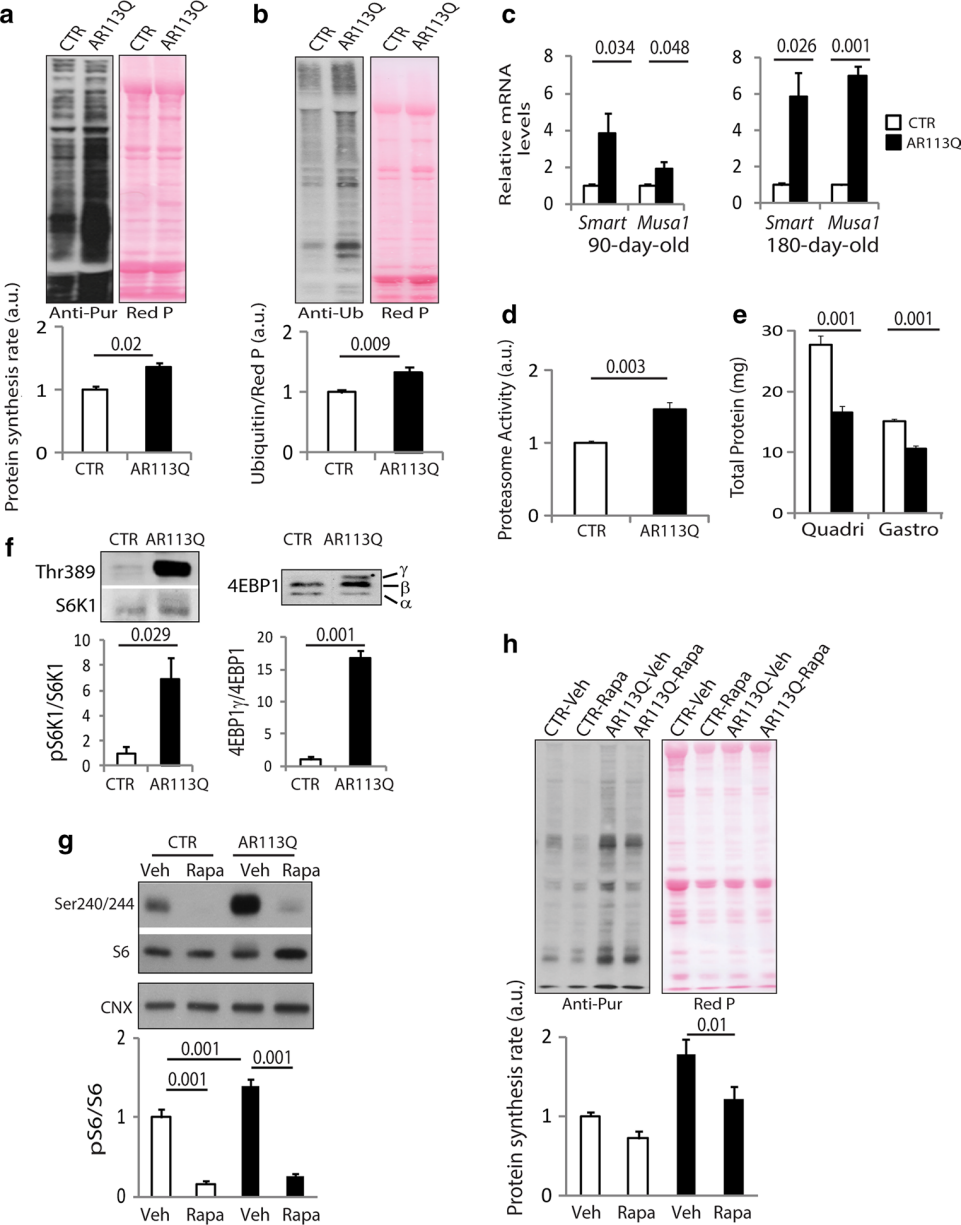
Protein turnover and mTOR signaling are enhanced in SBMA glycolytic muscles. **a** Western blotting analysis of the rate of new protein synthesis in the quadriceps of 90-day-old AR113Q and CTR (wild type) mice intraperitoneally injected with the aminoacyl-tRNA analog puromycin. Puromycin incorporation was detected with anti-puromycin (anti-pur) antibody, and Red Ponceau (*Red* *P*) was used as loading control. Graph shows mean ± sem, *n* = 3–5 mice. **b** Western blotting analysis of protein ubiquitination in the quadriceps of 90-day-old AR113Q and CTR (wild type) mice. Ubiquitinated proteins were detected with anti-ubiquitin (anti-Ub) antibody. Graph shows mean ± sem, *n* = 4–6 mice. **c** Real-time PCR analysis of the indicated E3 ubiquitin ligase transcript levels normalized to actin in the quadriceps of AR113Q and CTR (wild type) mice. Graphs show mean ± sem, *n* = 4–6 mice. **d** Proteasome activity measured in the quadriceps of 90-day-old AR113Q and CTR (wild type) mice. Graph shows mean ± sem, *n* = 4 mice. **e** Total protein content in quadriceps (quadri) and gastrocnemius (gastro) muscles of 90-day-old AR113Q and CTR (wild type) mice. Graph shows mean ± sem, *n* = 4 mice. **f** Western blotting analysis of S6K1 and 4EBP1 phosphorylation and expression levels in the quadriceps of 90-day-old AR113Q and CTR (AR21Q) male mice. Graphs show mean ± sem, *n* = 3 mice. **g** Western blotting analysis of phosphorylated and total S6 levels in the quadriceps of AR113Q and CTR (wild type) mice treated with vehicle (Veh) or rapamycin (Rapa). Graph shows mean ± sem, *n* = 6–8 mice. Calnexin (CNX) was used as loading control. **h** Western blotting analysis of the rate of new protein synthesis in the quadriceps of AR113Q and CTR (wild type) mice treated as in **g**. Graph shows mean ± sem, *n* = 6–7 mice

Muscle homeostasis is maintained by a fine balance between protein synthesis and degradation, two processes that simultaneously occur in muscle [100]. We hypothesized that the metabolic changes detected in SBMA muscle alter protein turnover. To assess the rate of new protein synthesis, we used in vivo surface sensing of translation (SUnSET) [92]. We intraperitoneally injected AR113Q and control mice with the antibiotic puromycin, and we analyzed the rate of new protein synthesis by Western blotting using an anti-puromycin antibody. The rate of new protein synthesis was increased by 35 and 73 % in the quadriceps muscle of 90- and 270-day-old AR113Q mice compared to age-matched control mice (Fig. 3a; Supplementary Fig. 10a); this increase was overt even upon 4 h of fasting (Supplementary Fig. 10b). We assessed protein degradation by analyzing ubiquitination of total protein lysates from quadriceps muscle of AR113Q and control mice. The amount of ubiquitinated proteins was increased by 1.33- and 1.84-fold in the quadriceps of 90- and 270-day-old AR113Q mice compared to control mice, respectively (Fig. 3b; Supplementary Fig. 10c). We observed increases in the expression of the E3 ubiquitin ligases, specific of muscle atrophy and regulated by transcription (*Smart*) and muscle ubiquitin ligase of the SCF complex in atrophy-1 (*Musa1*) (Fig. 3c) [56, 88], as well as increased proteasome activity starting at 90 days of age in the quadriceps muscle of AR113Q mice, further indicating enhanced protein degradation in the muscle of SBMA mice (Fig. 3d; Supplementary Fig. 10d). Notably, the net amount of protein content in the quadriceps and gastrocnemius muscles of AR113Q mice was reduced at 90 and 270 days of age (Fig. 3e; Supplementary Fig. 10e). These alterations were not detected in soleus (Supplementary Fig. 11a, b). These results show that protein turnover (protein synthesis and breakdown) is increased in SBMA glycolytic, but not oxidative muscles, and that the increased rate of protein degradation is not compensated by the increased rate of protein synthesis.

### Enhanced mTOR signaling is responsible for the increased rate of protein synthesis in SBMA glycolytic muscles

A key regulator of protein turnover in muscle is mTOR, a sensor of cellular metabolism and a regulator of glycolysis and lipid biosynthesis [30]. mTOR stimulates protein synthesis through phosphorylation of the eukaryotic translation initiation factor 4E binding protein 1 (4EBP1) and S6 kinase 1 (S6K1), which in turn phosphorylates the ribosomal protein S6. mTOR signaling was increased in the quadriceps, but not spinal cord, of 90- and 270-day-old AR113Q male, and not female, mice (Fig. 3f; Supplementary Fig. 12a). S6K1 phosphorylation was increased also in gastrocnemius and TA, which show atrophy, but not soleus, which is spared in this mouse model of SBMA (Supplementary Fig. 12b); the increase in mTOR signaling preceded atrophy, as it was detected as early as 40 days of age (Supplementary Fig. 12c). Enhanced mTOR signaling was not associated with increased expression of AR in male AR113Q mice compared to control mice (Supplementary Fig. 12a). Importantly, we did not detect alterations in the phosphorylation levels of Akt and its downstream effectors glycogen synthase kinase 3β (GSK3β) and forkhead box O transcription factors (FOXOs), suggesting that mTOR activation is independent of Akt. To assess whether enhanced mTOR signaling is responsible for the increased rate of new protein synthesis in SBMA muscle, we administered rapamycin (4 mg/kg/day) to AR113Q and control mice via intraperitoneal injections every day for 20 days. Rapamycin treatment reduced S6 phosphorylation (Fig. 3g) and decreased the rate of new protein synthesis by 28 and 32 % in control and AR113Q mice, respectively (Fig. 3h). Importantly, rapamycin decreased Akt phosphorylation in muscle, the body weight of AR113Q mice starting from 16 days of treatment, without altering food intake, and testis size in both AR113Q and control mice (Supplementary Fig. 13a–d). Rapamycin did not modify skeletal muscle mass (Supplementary Fig. 13e). Taken together, these results indicate that mTOR signaling is enhanced prior to atrophy, and it increases the rate of new protein synthesis in SBMA glycolytic muscles.

### Metabolic alterations in muscle precede denervation, mitochondrial depolarization and autophagy induction, and correlate with PGC1α expression

**Fig. 4:**
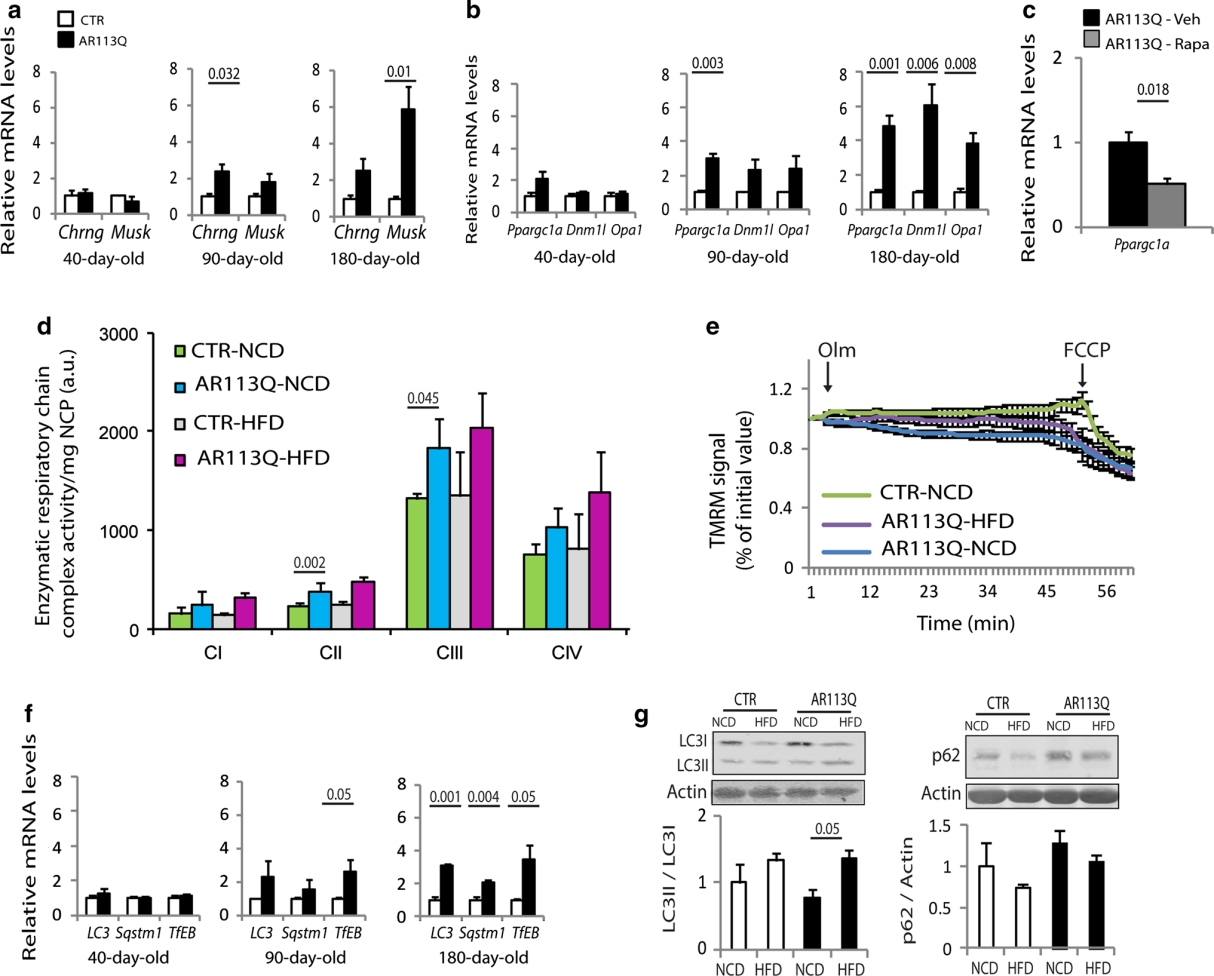
PGC1α expression is induced by mTOR and precedes denervation and autophagy activation in SBMA muscle. **a**–**c** Real-time PCR analysis of the indicated gene transcript levels normalized to actin in the muscle of AR113Q and CTR (wild type) mice. Graphs show mean ± sem, *n* = 4–6 mice. *Veh* vehicle, *Rapa* rapamycin. **d** Mitochondrial complex (C) I, II, III, and IV activity measured in the quadriceps of 180-day-old AR113Q and CTR (wild type) mice fed a normal chow diet (NCD) and a high-fat diet (HFD) and normalized to mitochondrial protein content. Graph shows mean ± sem, *n* = 4 mice. *NCP* non-collagen proteins. **e** Mitochondrial membrane depolarization measured in fibers isolated from flexor digitorum brevis of 180-day-old mice fed either an NCD or an HFD. Graph shows mean ± sem, *n* = 3–4 mice, 10 fibers. *Olm* oligomycin, *FCCP* protonophore carbonyl cyanide *p*-trifluoromethoxyphenylhydrazone TMRM, tetramethyl rhodamine methyl ester. **f** Real-time PCR analysis of the indicated gene transcript levels normalized to actin in the muscle of 180-day-old AR113Q and CTR (wild type) mice. Graphs show mean ± sem, *n* = 4–6 mice. **g** Western blotting analysis of LC3I and II and p62 in the quadriceps muscle of 180-day-old AR113Q and CTR (wild type) mice fed an NCD and an HFD. Graphs show mean ± sem, *n* = 4–6 mice

Denervation may cause metabolic alterations in muscle associated with atrophy and loss of fast-glycolytic fibers [64]. Therefore, we investigated whether the metabolic changes detected in quadriceps result from denervation in SBMA knock-in mice (Fig. 4a). The transcript levels of two denervation markers, acetylcholine receptor subunit gamma (*Chrng*) and muscle-specific kinase (*Musk*), were upregulated by 90 days of age, but not at 40 days of age, when the metabolic changes were started to be detected. Moreover, these denervation markers were not upregulated in the soleus of 180-day-old AR113Q mice (Supplementary Fig. 14a). These results indicate that the metabolic alterations that occur in SBMA glycolytic muscles precede denervation and are not initiated, rather maybe contributed at later stages of disease, by pathogenic processes occurring in motor neurons.

mTOR controls mitochondrial gene expression through modulation of PGC1α (*Ppargc1a*) function [20]. PGC1α is a master regulator of muscle metabolism, and when overexpressed it shifts muscle metabolism towards oxidative phosphorylation [47]. PGC1α expression correlated with disease progression in the quadriceps, but not soleus of AR113Q mice (Fig. 4b; Supplementary Fig. 14b). In quadriceps, PGC1α induction showed a trend for significance (*P* = 0.08) at 40 days of age, suggesting that its dysregulation occurs very early in disease pathogenesis, with dysregulation increasing with disease progression. Importantly, rapamycin treatment decreased PGC1α transcript levels, indicating that in SBMA muscle PGC1α expression is regulated by mTOR (Fig. 4c). PGC1α promotes mitochondrial biogenesis [104]. Markers of mitochondria fission and fusion, such as dynamin-related protein 1 (*Dnm1* *l*, Drp1) and opticatrophy gene 1 (*Opa1*), were upregulated in the quadriceps, but not soleus of AR113Q mice (Fig. 4b; Supplementary Fig. 14b). Moreover, the activity of mitochondrial complexes was increased in the quadriceps of AR113Q mice (Fig. 4d; Supplementary Fig. 15a). Mitochondrial abnormalities have been reported in SBMA [79]. We measured mitochondrial membrane potential in myofibers isolated from the fast-glycolytic flexor digitorum brevis muscle upon treatment with the F_1_F_0_-ATPase blocker, oligomycin (Fig. 4e; Supplementary Fig. 15b). We found that the number of fibers with mitochondria depolarized by oligomycin was significantly (*P* < 0.05) increased by 40 % in 90- and 180-day-old AR113Q mice. These data indicate that despite the increased beta oxidative metabolism, mitochondria are dysfunctional in SBMA muscle and are a potential source of oxidative stress.

Mitochondrial depolarization and accumulation of reactive oxygen species represent a signal for autophagy activation [90]. The autophagy markers microtubule-associated protein 1A/1B-light chain 3 (LC3) and sequestosome 1 (*Sqstm1*, p62) are upregulated in the muscle of AR113Q mice, indicating enhanced autophagy [16, 83]. The transcript levels of these autophagy markers were increased in the quadriceps, but not soleus, of 180-day-old AR113Q mice (Fig. 4f; Supplementary Fig. 14c). Upregulation of these autophagy markers in quadriceps was preceded by transcriptional induction of *TfEB*, the master regulator of autophagy gene expression [87]. To monitor autophagy flux, we measured the soluble mature form of LC3, namely LC3I, the membrane-bound form, LC3II, which accumulates upon autophagosome formation, and p62, which accumulates upon inhibition of autophagy flux (Fig. 4g; Supplementary Fig. 16a–c). Both LC3I and II increased at late stages of disease (180 and 270 days of age), and the ratio LC3II/LC3I did not change over the progression of disease, whereas p62 significantly accumulated in 270-day-old mice, suggesting defects in autophagy flux at very late stages of disease. These data indicate that metabolic changes in SBMA muscle precede TFEB upregulation and autophagy induction.

### An HFD ameliorates the phenotype of knock-in SBMA mice

**Fig. 5:**
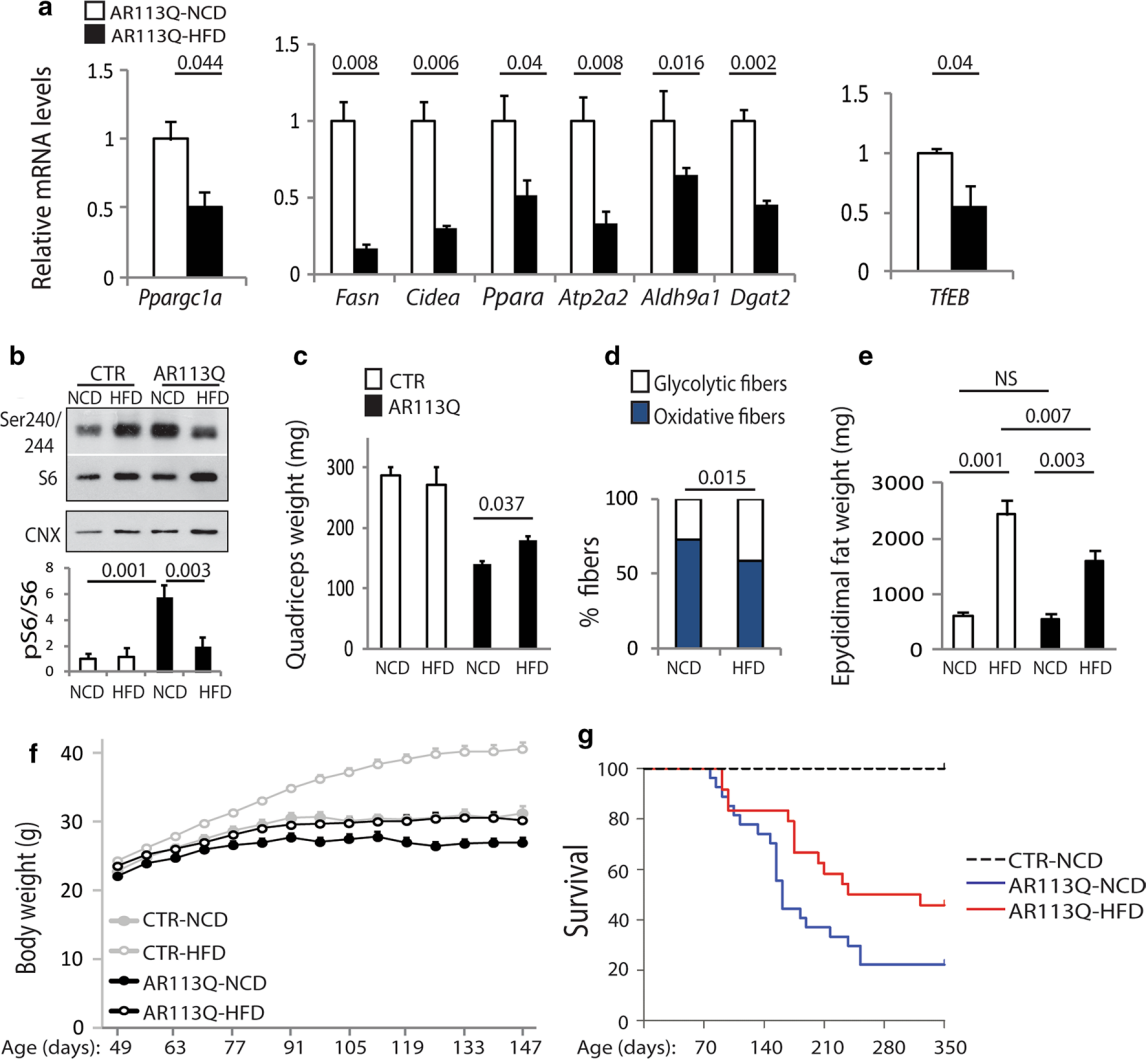
An HFD ameliorates the phenotype of SBMA knock-in mice. **a** Real-time PCR analysis of the transcript levels of selected genes in the muscle of 180-day-old AR113Q mice fed as indicated and normalized to beta-glucuronidase and actin. Graphs show mean ± sem, *n* = 5 mice. **b** Western blotting analysis of phosphorylated and total S6 in the quadriceps of 180-day-old AR113Q and CTR (wild type) mice fed either NCD or HFD. Calnexin (CNX) was used as loading control. Graph shows mean ± sem, *n* = 4–8 mice. **c** Analysis of quadriceps wet weight in AR113Q and CTR (wild type) mice fed as indicated. Graph shows mean ± sem, *n* = 4–8 mice. **d** NADH staining analysis in the quadriceps of 180-day-old AR113Q mice fed either NCD or HFD. Graph shows mean ± sem, *n* = 3–4 mice. **e** Analysis of epididymal fat weight in 180-day-old AR113Q and CTR (wild type) mice fed as indicated. Graph shows mean ± sem, *n* = 3–9 mice. **f** Body weight analysis of AR113Q and CTR (wild type) fed either NCD or HFD. Graph shows mean ± sem, *n* = 18–21 mice. **g** Kaplan–Meier analysis of survival (*n* = 32 CTR-NCD, 29 CTR-HFD, 27 AR113Q-NCD, and 24 AR113Q-HFD)

mTOR and PGC1α can be inhibited by feeding mice an HFD [6, 97]. Therefore, we administered AR113Q and control mice either an HFD or the NCD (Supplementary Fig. 17). Treatment was started at 40 days of age. The HFD reduced the changes in gene expression detected in SBMA muscle (Supplementary Fig. 18a, Tables 3–5), including *Ppargc1a* (PGC1α), genes involved in lipid metabolism, and *TfEB* (Fig. 5a), it reduced mitochondrial membrane depolarization by 50 % without altering the activity of mitochondrial complexes (Fig. 4d, e), it increased LC3 lipidation (Fig. 4g), and it restored the activation of mTOR signaling to normal levels (Fig. 5b). The HFD did not affect the levels of expression of polyglutamine-expanded AR (Supplementary Fig. 18b). The HFD increased the wet weight of quadriceps (Fig. 5c) but not gastrocnemius (Supplementary Fig. 19a), and reduced the number of slow-oxidative fibers by 20 % in both quadriceps and gastrocnemius muscles of SBMA mice (Fig. 5d; Supplementary Fig. 19b, c), it increased the mean CSA of oxidative fibers in the quadriceps and gastrocnemius by 11 and 7 %, respectively, whereas it did not affect the CSA of glycolytic fibers (Supplementary Fig. 20a, b). In CTR mice, the HFD caused glucose intolerance and insulin resistance (Supplementary Fig. 8b, c), signs of metabolic syndrome that normal mice develop upon exposure to HFD [35]. However, the HFD did not elicit signs of metabolic syndrome in AR113Q mice. The HFD increased fat deposition by 2.9- and 3.9-fold in AR113Q and CTR mice, respectively (Fig. 5e). Control mice fed the HFD exhibited a progressive increase in body weight (*P* = 0.00001) from 70 days of age compared to control mice fed the NCD (Fig. 5f; Supplementary Fig. 21a, b). AR113Q mice also showed a significant (*P* = 0.00001) increase in body weight starting from 84 days of age and were indistinguishable from control mice fed the NCD. The body weight of control and AR113Q mice fed the HFD versus NCD was increased by 1.84- and 1.49-fold, respectively, indicating that AR113Q are partially resistant to diet-induced obesity, despite similar food intake and locomotor activity (Supplementary Fig. 21c, d). AR113Q mice showed reduced performance on an accelerating rotarod (Supplementary Fig. 21e). The performance of control mice fed the HFD was significantly decreased, as expected, whereas that of AR113Q mice was restored to normal. The HFD also increased the force of gastrocnemius (Supplementary Fig. 21f). Importantly, the HFD extended the median life span of AR113Q mice from 161 days to 322 days (χ2LR = 3.955, *P* = 0.047; Gehan–Breslow: *P* = 0.046) (Fig. 5g). These results show that the HFD attenuates disease manifestations in knock-in SBMA mice.

### Metabolic alterations in glycolytic muscles of SBMA patients

**Fig. 6:**
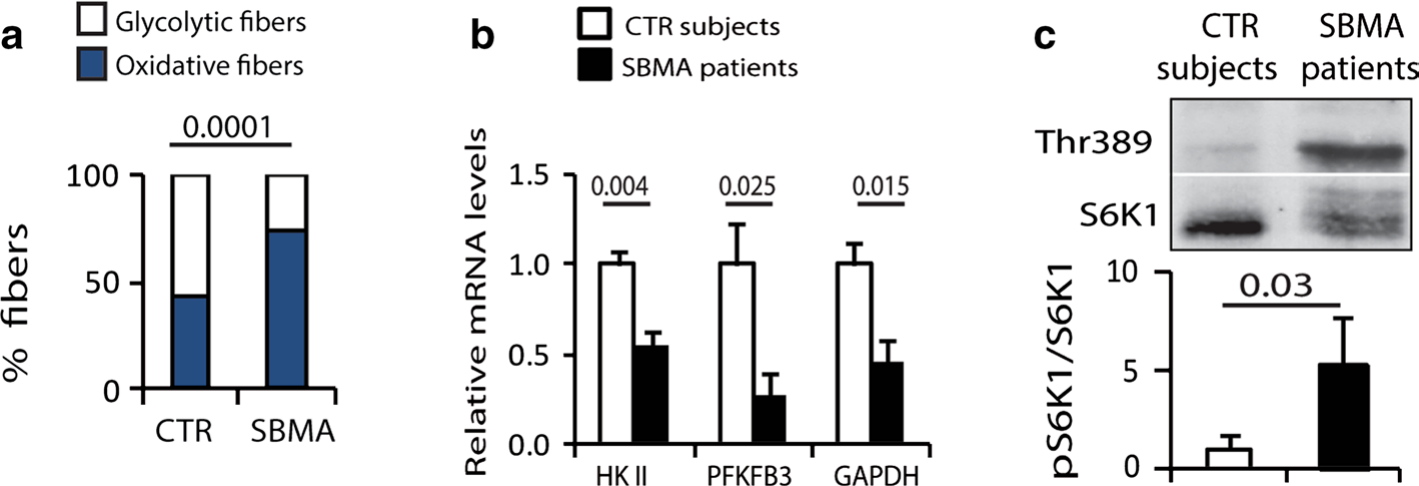
Impaired glycolysis, fiber-type switch, and enhanced mTOR signaling in the muscle of SBMA patients. **a** NADH staining of muscle biopsy specimens from SBMA patients and CTR subjects. Graph shows mean ± sem, *n* subjects = 4 SBMA and 6 CTR, *n* fibers = 1964 SBMA and 1924 CTR. **b** Real-time PCR analysis of glycolytic gene transcript levels normalized to beta-glucuronidase in the muscle of SBMA patients and CTR subjects. Graph shows mean ± sem, *n* = 5 SBMA and 5 CTR subjects. **c** Western blotting analysis of phosphorylated and total S6K1 in the muscle of SBMA patients and CTR subjects. Graph shows mean ± sem, *n* = 7 CTR and 14 SBMA subjects

We next assessed whether metabolic alterations also occur in the muscle of SBMA patients. The number of NADH-positive fibers was increased by 1.7-fold in the muscle of SBMA patients compared to normal subjects (Fig. 6a). The expression of the key glycolytic genes, *HkII*, *Pfkfb3*, and *Gapdh*, was significantly decreased in the glycolytic muscles of SBMA patients, suggesting that glycolysis is impaired in the muscle of SBMA patients (Fig. 6b). Finally, the phosphorylation of the mTOR downstream target S6K1 was augmented in the muscle of SBMA patients compared to control subjects, indicating that mTOR signaling is enhanced in SBMA patients (Fig. 6c). These results show metabolic alterations in the muscle of SBMA patients, thereby supporting the biological relevance of our findings in pathogenesis.

## Discussion

That primary cell-autonomous degenerative processes occur in SBMA muscle is supported by multiple lines of evidence [84], including the observations that abnormalities were detected in cultured myotubes isolated from the quadriceps of SBMA patients [52], modulation of expression of non-expanded as well as polyglutamine-expanded AR in muscle modifies disease [17, 59, 77], and intervention to reduce the accumulation of polyglutamine-expanded AR selectively in muscle is beneficial in SBMA mice [70]. What is less obvious is how expression of polyglutamine-expanded AR causes damage to muscle, and how this in turn contributes to the metabolic alterations found in SBMA patients [75]. Skeletal muscle plays a central role in the regulation of glucose and fatty acid metabolism [25]. Skeletal muscle metabolism is dependent on fiber-type composition, with slow-twitch oxidative fibers using lipids as their main source of energy, and fast-twitch glycolytic fibers using glucose. We observed metabolic changes characterized by a glycolytic-to-oxidative switch in muscle in both knock-in mice and SBMA patients. In SBMA knock-in mice, this fiber-type switch preceded atrophy. A similar glycolytic-to-oxidative shift in fiber-type composition was overt in the quadriceps muscle of transgenic mice ubiquitously expressing polyglutamine-expanded AR, and it was attenuated by knockdown of mutant AR solely in muscle [45]. A glycolytic-to-oxidative fiber-type switch has also been observed in AR knock-out mice, suggesting that polyglutamine expansion causes this phenomenon in muscle mainly through a loss of function mechanism [50]. Notably, in the knock-in SBMA mice the degree of atrophy of glycolytic fibers was higher than that of oxidative fibers. Because similar changes in muscle homeostasis occur during aging [39], these observations suggest that SBMA muscle may undergo premature aging, and that a genetic interaction between aging and expression of polyglutamine-expanded AR may contribute to SBMA pathogenesis. In addition to the loss of function, there is evidence that also gain of native AR function contributes to SBMA pathogenesis [63, 89]. Indeed, overexpression of non-expanded AR solely in muscle caused severe muscle atrophy and premature death [59], and overexpression of polyglutamine-expanded AR in either skeletal muscle or neurons reduced the size of fast-glycolytic fibers and induced an oxidative-to-glycolytic fiber-type shift in extensor digitorum longus [77]. Interestingly, the levels of expression of polyglutamine-expanded AR correlated with the degree of atrophy, supporting the idea that expression of polyglutamine-expanded AR in muscle exerts cell-autonomous, dose-dependent toxic effects in muscle. Moreover, our findings suggest that dysregulation of androgen signaling in muscle alters muscle and body metabolism, thereby contributing to SBMA pathogenesis.

Our lipidomic analysis revealed increased levels of lysophosphatidylcholines, lysophosphatidylethanolamines, ceramides, diglycerides, and polyunsaturated fatty acids, and decreased levels of species enriched in polyunsaturated lipids and phosphatidylglycerol. By microarray analysis, we observed augmented expression of genes involved in lipid biosynthesis and lipolysis. Consistent with the observation that glycolytic muscles undergo a shift towards oxidative metabolism, these findings suggest that lipid synthesis and turnover are enhanced in SBMA glycolytic, but not oxidative muscles. Another interesting feature that characterizes SBMA muscle is the reduction of expression and activity of glycolytic enzymes, which also occurs during aging [4]. Notably, changes in expression of glycolytic genes preceded those in lipid genes, suggesting that altered glycolysis contributes to the shift towards oxidative phosphorylation. This finding raises the question of whether polyglutamine expansion in AR directly hampers the expression of key glycolytic genes, such as *HkII* and *Pfkfb*, which are direct target of AR [60]. Moreover, the expression of the glucose transporter GLUT3 was decreased in the muscle of AR113Q mice. GLUT3 has a higher affinity for glucose and at least a fivefold greater transport capacity than other glucose transporter family members, such as GLUT1, GLUT2 and GLUT4. The decrease in GLUT3 expression is consistent with the glycolytic-to-oxidative fiber-type switch detected in SBMA muscle and suggests that glucose uptake is defective in SBMA glycolytic muscles. Interestingly, the expression of GAPDH was decreased at late stages of disease also in soleus, which does not develop atrophy and does not show signs of denervation. It is possible that polyglutamine expansion in the AR directly alters the expression of glycolytic genes, which is detrimental in muscles that are mostly composed of glycolytic fibers, such as quadriceps and gastrocnemius. This effect also occurs to a lower extent in muscles that are mostly composed of oxidative fibers, but in this case with limited consequences on muscle metabolism. Further investigation is required to address whether altered glycolysis underlies or contributes to the metabolic shift in glycolytic muscles toward oxidative phosphorylation.

Glycolytic-to-oxidative fiber-type switch has also been reported in amyotrophic lateral sclerosis (ALS) [68]. Similar to SBMA, glycolytic muscles are more severely affected in transgenic mouse models of superoxide dismutase 1 (SOD1)-linked ALS [68, 74] and in ALS patients [26], and this is likely due to selective vulnerability of fast-fatiguable motor neurons [74]. Notably, the phenotype of mutant SOD1-expressing mice is different from that of SBMA mice. Indeed, while mutant SOD1-expressing mice develop paralysis of posterior limbs, SBMA mice do not develop paralysis even at late stages of disease, indicating that the degenerating motor neurons maintain their ability to control muscle contraction to some extent. In SBMA muscle, upregulation of denervation markers was detected after metabolic changes, indicating that, in contrast to ALS mice, in SBMA mice these alterations precede denervation. Importantly, the motor neuron number and morphology of neuromuscular junctions are preserved in knock-in SBMA mice [40], further supporting that the metabolic changes detected in SBMA glycolytic muscles are not induced by denervation at early stages of disease.

SBMA muscle was characterized by increased rates of protein synthesis and degradation in glycolytic muscles. Protein synthesis was induced by enhanced activation of mTOR signaling, which occurred selectively in atrophic muscles, was an early event, and was also observed in the muscle of SBMA patients. Dysregulation of mTOR signaling is emerging as a key component of neurodegenerative disease pathogenesis. mTOR signaling is upregulated in the muscle of an HD mouse model [95] and in the cortex of mice overexpressing TAR DNA-binding protein 43 (TDP43) [102]; in contrast, it is downregulated in the muscle of a mouse model of valosin-containing protein-associated inclusion body myopathy [14], in the striatum of HD patients and mice [44], and in the spinal cord of transgenic mice expressing mutant SOD1 [109]. mTOR activation can be elicited in response to denervation and excessive accumulation of amino acids released from increased protein breakdown. However, in SBMA muscle mTOR activation preceded denervation. It remains to be elucidated why mTOR is upregulated in SBMA muscle. We noticed that mTOR activation correlated with the physiological increase of serum testosterone levels that occurs at approximately 30 days of age in mice. This observation suggests that activation of AR is required for mTOR induction, and the crosstalk existing between androgen and mTOR signaling may lead to enhancement of mTOR function and muscle hypertrophy in physiological conditions [5]. In SBMA, polyglutamine expansion in the AR may result in aberrant and sustained activation of mTOR that in turn can contribute to muscle atrophy during disease progression. Prolonged activation of mTORC1 causes a late-onset myopathy characterized by altered autophagy [12]. As a matter of fact, autophagy was induced in SBMA muscle and altered at very late stages of disease. Although further experimental evidence is required to assess the role of mTOR in SBMA muscle, it is possible that enhancement of mTOR signaling at early stages of disease is compensatory to the increased need of protein synthesis and breakdown occurring in response to the glycolytic-to-oxidative metabolic switch in muscle, but prolonged activation of mTOR may turn to be detrimental at later stages of disease. mTOR forms two complexes, mTOR complex 1 (mTORC1), which regulates cell metabolism, protein synthesis and autophagy, and mTOR complex 2 (mTORC2), which mainly controls cell proliferation and survival. In SBMA muscle, mTORC1 is activated very early, before induction of denervation, and it increases the rate of new protein synthesis. mTORC1 is sensitive to rapamycin. Rapamycin exerts beneficial effects in several neurodegenerative diseases. In a pharmacological model of Parkinson’s disease (PD), rapamycin treatment decreased dopaminergic neuron loss by inhibiting the expression of mTORC1-induced pro-apoptotic genes [51], and by activating autophagy [22]. Induction of autophagy and clearance of aggregates underlie the beneficial effects of rapamycin in mouse models of Alzheimer’s disease (AD) [10, 98], HD [80], SCA3 [55], and PD [19], and in mice overexpressing TDP43 modeling frontotemporal dementia [102]. However, rapamycin worsened the phenotype of transgenic mice expressing either mutant SOD1 [109] or mutant valosin-containing protein (VCP) [14]. Rapamycin treatment resulted in exacerbation of body weight loss in SBMA knock-in mice, similar to what has been observed in other pathological conditions (reviewed by [53]). It is noteworthy that prolonged treatment with rapamycin inhibits mTORC2 and Akt in myoblast cells [86], and as shown here in SBMA muscle. Because Akt-mediated phosphorylation of polyglutamine-expanded AR promotes degradation and reduces toxicity, it is possible that rapamycin-induced deterioration of phenotype in SBMA mice results from Akt inactivation in muscle [69, 70, 89].

mTOR controls cell metabolism through regulation of PGC1α function [20]. PGC1α is upregulated selectively in SBMA glycolytic muscles. Different from SBMA, PGC1α is downregulated in the muscle of mutant SOD1-linked ALS mice [68], and its overexpression selectively in muscle attenuated motor dysfunction, even if it had no effect on survival [21]. While in ALS muscle the glycolytic-to-oxidative fiber-type switch is independent of PGC1α [68], our results show that in SBMA muscle PGC1α is progressively upregulated selectively in atrophic muscles following disease progression, thereby suggesting a role for this nuclear receptor co-regulator in SBMA pathogenesis. Several transcriptional co-regulators that interact with AR and other nuclear receptors play an important role in controlling muscle fiber-type composition. For instance, muscle-specific ablation of nuclear receptor co-activators, such as Sox6 and Mediator 1, or co-repressors, such as RIP140, lead to a glycolytic-to-oxidative fiber-type switch [13, 47, 76, 93]. The upregulation of PGC1α detected in SBMA muscle was decreased by treatment of the mice with rapamycin, indicating that PGC1α is induced by mTOR. Muscle-specific overexpression of PGC1α leads to a glycolytic-to-oxidative fiber-type switch [47], whereas loss of PGC1α function has the opposite effect [37]. Moreover, PGC1α controls the expression of genes involved in oxidative phosphorylation [61]. These observations suggest a model whereby polyglutamine-expanded AR leads to mTOR activation, which in turn leads to PGC1α induction and glycolytic-to-oxidative fiber-type switch. PGC1α levels and mTOR activation can be modulated by a diet enriched with lipids [6, 97]. Importantly, feeding SBMA mice an HFD decreased mTOR signaling activation, reduced the expression of PGC1α as well as of genes involved in oxidative phosphorylation, and attenuated the glycolytic-to-oxidative metabolic fiber-type switch.

PGC1α promotes mitochondrial biogenesis [104]. Consistent with the glycolytic-to-oxidative fiber-type switch and the upregulation of PGC1α, genes involved in mitochondrial dynamics were induced, and the activity of citrate synthase and the mitochondrial respiratory complexes was increased in SBMA muscle. Mitochondrial homeostasis is altered in SBMA [79], and mitochondrial membrane potential was decreased by oligomycin in glycolytic SBMA muscles. While the HFD did not affect mitochondrial complex activity, it decreased mitochondrial membrane depolarization induced by oligomycin, indicating that the HFD improves mitochondrial quality control. Mitochondrial dysfunction is often associated with induction of autophagy [90]. Autophagy is induced in the muscle of SBMA knock-in mice [16, 108], and our results show that autophagy flux is altered at late stages of disease. Notably, TFEB is induced in SBMA muscle, leading to upregulation of expression of several autophagy target genes [16]. In neurons, TFEB has been shown to work as a co-factor of AR, whose function is diminished by polyglutamine expansion [18]. It is possible that in muscle polyglutamine expansion in AR leads to a gain of TFEB function, resulting in enhanced autophagy. Although TFEB is negatively regulated by mTOR [94], we detected concurrent mTOR and TFEB activation in SBMA glycolytic, but not oxidative muscles, providing evidence that in disease conditions the two signaling pathways can be simultaneously activated. Importantly, the HFD reduced the expression of TFEB, linking TFEB to metabolism. Interestingly, an HFD attenuated autophagy defects and muscle atrophy caused by muscle-specific knock-out of histone deacetylases I and II, supporting the idea that HFD rescues autophagy alterations and muscle degeneration [62]. Consistent with this observation, the HFD decreased the levels of LC3I while increasing the levels of LC3II, suggesting a positive effect on autophagosome formation and autophagy flux.

Progressive muscle atrophy and body weight loss are prognostic factors for several neurodegenerative diseases, such as AD [54], HD [85], PD [1, 7], and ALS [27]. Body weight loss can result from reduced energy intake, increased energy expenditure, or both. Weight loss cannot be explained by decreased food intake in these conditions [85, 101]. Rather, neurodegenerative disorders are often associated with a hypermetabolic state [28, 58], suggesting that altered energy homeostasis contributes to pathogenesis [73]. These systemic metabolic deficits are characterized by altered lipid (cholesterol and fatty acid) metabolism, as reported in HD [9], and AD [29, 46]. Interestingly, male ALS patients have hypolipidemia [105], and decreased levels of low-density lipoprotein have been shown to correlate with respiratory impairment [15]. High levels of serum cholesterol and triglycerides correlate with longer survival, further highlighting the relevance of lipid metabolism in ALS [32]. Moreover, epidemiological studies showed that a higher body fat content is associated with a lower risk for ALS [36, 48], whereas a high-carbohydrate/low-fat diet is associated with an increased risk for ALS [66]. A beneficial effect of high-calorie diet has been proven in patients suffering from HD and ALS [31, 85, 101, 103], as well as in mice expressing either mutant SOD1 [33] or mutant VCP [49]. Notably, an HFD has been shown to stabilize body weight loss in ALS patients [31]. Recently, in a double-blind, placebo-controlled clinical trial, a high-carbohydrate diet, but not an HFD, ameliorated some aspects of disease in ALS patients [103]. In this trial, treatment was started after about 20 % body weight loss, and it remains to be established whether an HFD can be beneficial if treatment is started at earlier stages of disease. Different from ALS patients, in SBMA patients the average serum lipid levels are on the upper range to normal, and low-density lipoprotein levels are elevated in some patients [23, 34, 75]. In knock-in SBMA mice, we did not detect serum lipid alterations. Feeding normal mice an HFD results in development of metabolic syndrome characterized by glucose intolerance, insulin resistance and obesity [35]. AR113Q mice neither developed signs of glucose intolerance and insulin resistance nor they became obese when fed the HFD. Moreover, the HFD increased the weight of fat pad in AR113Q mice to a lesser extent compared to control mice. These results support the idea that SBMA mice require a higher caloric intake compared to control mice. Indeed, skeletal muscle represents 30 % of body weight, and a metabolic change in muscle towards oxidative phosphorylation is expected to impact body metabolism with increased need for fatty acids. Therefore, the effect of chronic exposure to HFD on metabolism leads to development of metabolic syndrome in control mice, but not SBMA mice, in which fatty acids are used by glycolytic muscles that have undergone a shift towards oxidative metabolism. However, before this approach can be translated to clinic, it is important to consider that an HFD may have undesired side effects in patients with serum hyperlipidemia.

Our results provide proof-of-principle that an HFD ameliorates muscle pathology, improves motor function, and extends life span of a mouse model of SBMA. The HFD ameliorated SBMA mouse phenotype by possibly affecting several tissues and metabolic pathways. In addition to muscle, the effect of diet may also come from adipose tissue. AR is highly expressed in adipose tissue, and the effect of polyglutamine expansion in AR on this peripheral tissue remains to be addressed. Our findings suggest an unprecedented role for adipose tissue in SBMA. Notably, androgen signaling mediated by the AR inhibits adipocyte differentiation and promotes lipolysis [65]. Adipose tissue controls whole body insulin sensitivity. Interestingly, pan-tissue knock-out of AR in mouse results in increased fat pad mass with normal insulin sensitivity and reduced body weight [78]. On the other hand, adipose tissue-specific knock-out of AR resulted in hyperinsulinemia in the absence of obesity, and when fed an HFD, the adipose tissue-specific AR knock-out mice developed obesity, hyperglycemia, and impaired insulin secretion. Another important aspect concerning adipose tissue and metabolism is the interplay between androgens and adipokine signaling [65]. High molecular weight adiponectin is expressed in white adipose tissue, increases insulin sensitivity, and is downregulated in obesity. Androgens inhibit the release of high molecular weight adiponectin, and castration or AR knock-out increased the serum levels of adiponectin. Further analysis will establish whether polyglutamine expansion in the AR alters adipokine signaling in SBMA. Our findings are consistent with a model whereby polyglutamine expansion in AR, in addition to conferring toxic gain of functions, leads to loss of AR function in muscle and possibly other tissues, such as adipose tissue. Some of these aspects were attenuated by the HFD, suggesting that this as a novel strategy in support to SBMA patients to modify disease onset, progression, and outcome.

## Electronic supplementary material

Below is the link to the electronic supplementary material. Additional details on Materials and Methods are provided in the Supplementary Information, together with 5 Supplementary Tables, 21 Supplementary Figures, and Supplementary References.

Supplementary material 1 (PDF 1916 kb)

Supplementary material 2 (XLSX 12 kb)

Supplementary material 3 (XLSX 21 kb)

Supplementary material 4 (XLSX 220 kb)

Supplementary material 5 (XLSX 9 kb)

Supplementary material 6 (XLSX 13 kb)
