# Supplementary material for: Glycolytic-to-oxidative fiber-type switch and mTOR signaling activation are early-onset features of SBMA muscle modified by high-fat diet
Source: Acta Neuropathol. 2016 Mar 12;132:127–44. doi: 10.1007/s00401-016-1550-4 (PMC4911374; doi:10.1007/s00401-016-1550-4)
Supplement: Supplementary file 1 — Supplementary material 1 (PDF 1916 kb) [file 401_2016_1550_MOESM1_ESM.pdf]

Glycolytic-to-oxidative fiber-type switch and mTOR signaling activation are early-onset features of SBMA muscle modified by high-fat diet

Acta Neuropathologica

Anna Rocchi[1#], Carmelo Milioto[1,2,3#], Sara Parodi[1,4], Andrea Armirotti[5], Doriana Borgia[6], Matteo Pellegrini[1,†], Anna Urciuolo[7], Sibilla Molon[7], Valeria Morbidoni[7], Manuela Marabita[8], Vanina Romanello[8], Pamela Gatto[9], Bert Blaauw[8], Paolo Bonaldo[7], Fabio Sambataro[10], Diane M. Robins[11], Andrew P. Lieberman[12], Gianni Sorarù[6], Lodovica Vergani[6], Marco Sandri[8,13], Maria Pennuto[1,2*]

[1]Department of Neuroscience and Brain Technologies, Istituto Italiano di Tecnologia, 16163 Genova, Italy
[2]Dulbecco Telethon Institute, Centre for Integrative Biology, University of Trento, 38123 Trento, Italy
[3]Dipartimento di Medicina Sperimentale, University of Genova, 16100 Genova, Italy
[4]Neurogenetics Branch, NINDS, National Institutes of Health, Bethesda, MD 20892, USA
[5]Drug Discovery and Development Department, Istituto Italiano di Tecnologia, 16100 Genova, Italy
[6]Department of Neurosciences, University of Padova, 35100 Padova, Italy
[7]Department of Molecular Medicine, University of Padova, 35131, Padova, Italy
[8]Venetian Institute of Molecular Medicine, Department of Biomedical Science, University of Padova, 35100 Padova, Italy
[9]High-Throughput Screening Core Facility, Centre for Integrative Biology, University of Trento, 38123 Trento, Italy
[10]Department of Experimental & Clinical Medical Sciences (DISM), University of Udine, 33100 Udine, Italy
[11]Department of Human Genetics, University of Michigan Medical School, Ann Arbor, MI 48109, USA
[12]Department of Pathology, University of Michigan Medical School, Ann Arbor, MI 48109, USA
[13]Telethon Institute of Genetic and Medicine, Pozzuoli, 80100 Napoli, Italy
[†]Current address: Department of Experimental Medicine (DIMES)—Biochemistry Section, University of Genova, Viale
Benedetto XV, 1–16132, Genova, Italy
# These two authors contributed equally to this work.

*Corresponding author:
Maria Pennuto
MPennuto@Dti.Telethon.it
Phone +39 0461 285215
Fax: +39 0461 283937

Table Legends

Supplementary Table 1.

List of primers used for real-time PCR analysis.

Supplementary Table 2.

Sheet 1: List of lipids enriched in AR113Q mice fed either the normal chow diet (AR113Q-NCD) or the high-fat diet (AR113Q-HFD) and CTR (wild type) mice fed the high-fat diet (CTR-HFD) compared to CTR mice fed the normal chow diet (CTR-NCD).

Fold change ≥ 1.50; p-value ≤ 0.001. RT= retention time; m/z = mass-to-charge ratio.

Sheet 2: List of lipids downregulated in AR113Q mice fed either the normal chow diet (AR113Q-NCD) or the high-fat diet (AR113Q-HFD) and CTR (wild type) mice fed the high-fat diet (CTR-HFD) compared to CTR mice fed the normal chow diet (CTR-NCD).

Fold change ≤ 0.50; p-value ≤ 0.001. RT= retention time; m/z = mass-to-charge ratio.

Supplementary Table 3.

Sheet 1: List of upregulated genes in AR113Q mice fed a normal chow diet (AR113Q-NCD) and AR113Q mice fed a high-fat diet (AR113Q-HFD) compared to CTR (wild type) mice fed the normal chow diet (CTR-NCD).

Fold change ≥ 1.5; p-value ≤ 0.05.

Sheet 2: List of downregulated genes in AR113Q mice fed a normal chow diet (AR113Q-NCD) and AR113Q mice fed a high-fat diet (AR113Q-HFD) compared to CTR (wild type) mice fed the normal chow diet (CTR-NCD).

Fold change ≤ 0.6; p-value ≤ 0.05.

Supplementary Table 4.

Markers of oxidative and glycolytic fibers upregulated and downregulated in AR113Q mice fed a normal chow diet (AR113Q-NCD) and AR113Q mice fed a high-fat diet (AR113Q-HFD) compared to CTR (wild type) mice fed the normal chow diet (CTR-NCD).

Fold change ≥ 1.5; p-value ≤ 0.05.

Fold change ≤ 0.6; p-value ≤ 0.05.

Supplementary Table 5.

Sheet 1: Gene set enrichment analysis (GSEA) of the transcriptome profile of AR113Q mice fed either a normal chow diet (AR113Q-NCD) or the high-fat diet (AR113Q-HFD) compared to CTR (wild type) mice fed a normal chow diet (CTR-NCD).

Top KEGG pathways (gene set) identified based on the genes differentially expressed in AR113Q-NCD and CTR-NCD mice (FDR q-val ≤ 0.25). The KEGG pathways rescued by the HFD mice are highlighted in blue. ES = enrichment score, NES = normalized enrichment score, NOM p-val = nominal p value, FDR q-val = False Discovery Rate, FWER p-val = family-wise error rate, RANK AT MAX = position in the ranked list at which the maximum enrichment score occurred.

Sheet 2: Gene set enrichment analysis (GSEA) of the transcriptome profile of AR97Q mice.

Top KEGG pathways (gene set) identified based on the genes differentially expressed in AR97Q and CTR mice fed a normal chow diet (FDR q-val ≤ 0.25) identified in a previous microarray analysis [3]. Pathways dysregulated in both AR113Q and AR97Q are highlighted in blue. ES = enrichment score, NES = normalized enrichment score, NOM p-val = nominal p value, FDR q-val = False Discovery Rate, FWER p-val = family-wise error rate, RANK AT MAX = position in the ranked list at which the maximum enrichment score occurred.

Supplementary Figures

Supplementary Figure 1

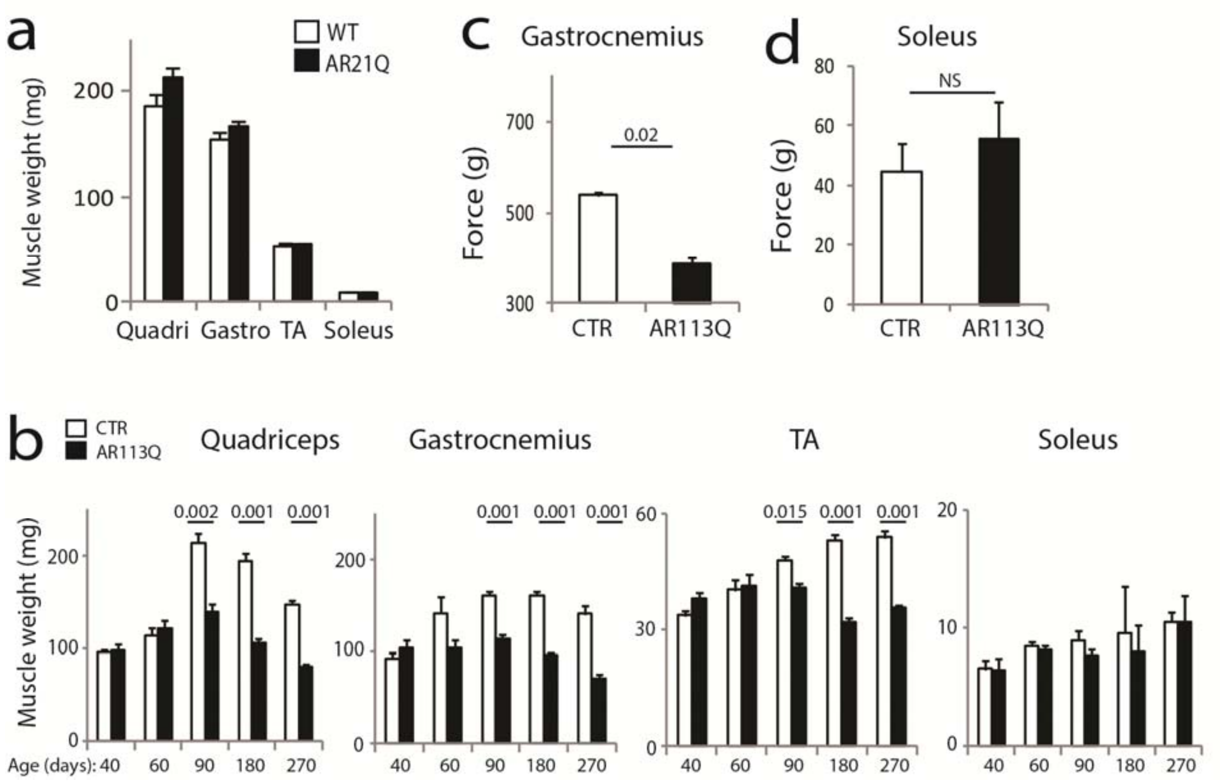

Supplementary Figure 1. The mass and force of glycolytic muscles is decreased in SBMA knock-in mice.

a) Muscle weight analysis in wild type (WT, C57Bl6J) and AR21Q mice. The wet weight of quadriceps (quadri), gastrocnemius (gastro), tibialis anterior (TA), and soleus was similar in 180-day-old WT and AR21Q mice. Graph shows mean ± sem, n = 5-11 mice.

b) Wet weight of the indicated skeletal muscles of AR113Q and control (CTR, wild type) mice analyzed at different ages. Graphs show mean ± sem, n = 3-10 mice.

c) *In vivo* force generation of gastrocnemius muscle measured in live 90-day-old AR113Q and CTR (wild type) mice. Tetanic force was decreased in AR113Q mice compared to CTR mice. Graph shows mean ± sem, n = 4 mice.

d) *Ex vivo* force generation of soleus muscle measured in 180-day-old AR113Q and CTR (wild type) mice. Tetanic force was similar in AR113Q mice compared to CTR mice. Graph shows mean ± sem, n = 5 mice.

Supplementary Figure 2

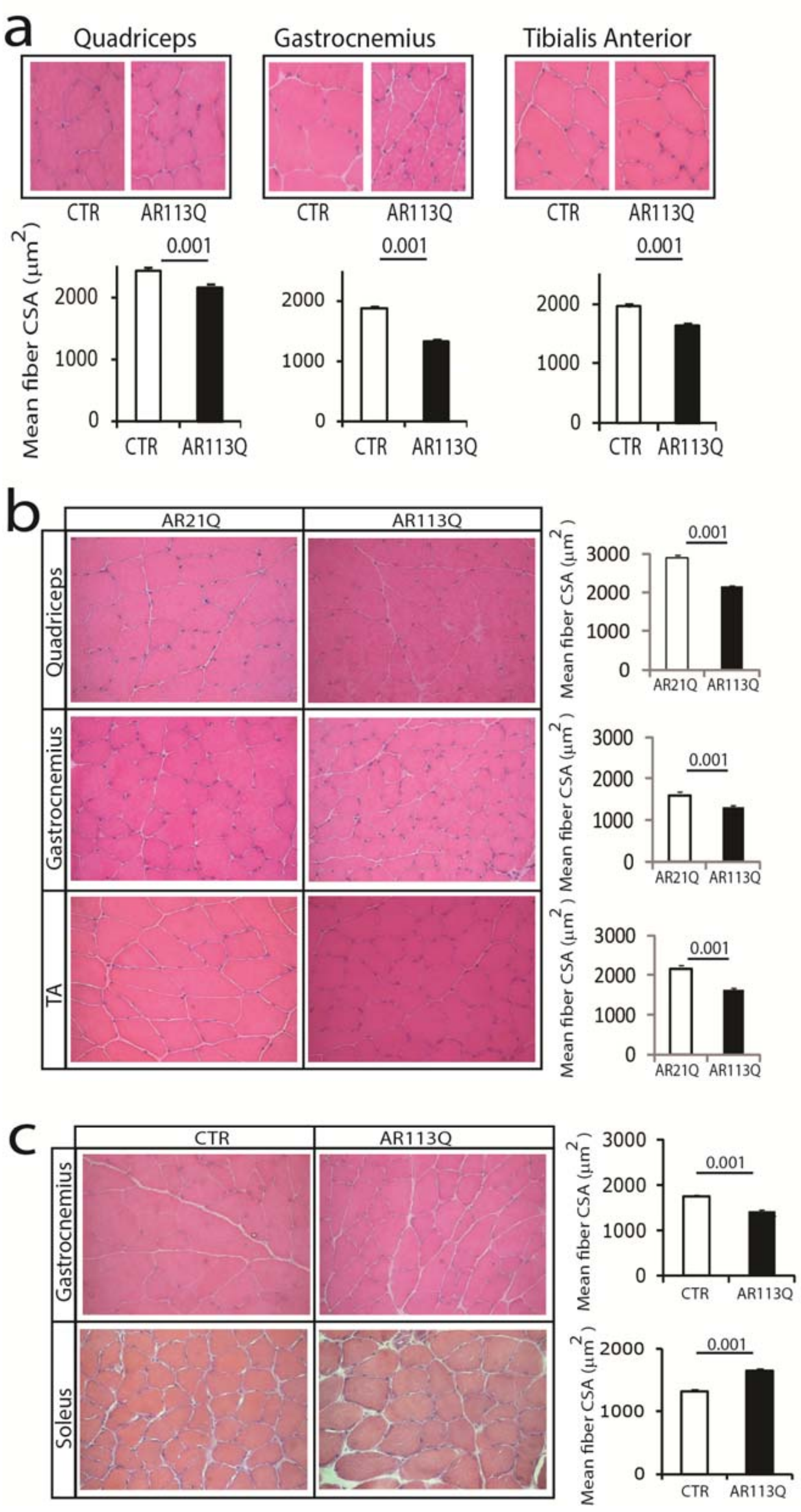

Supplementary Figure 2. The cross-sectional area of glycolytic muscles is decreased in SBMA knock-in mice.

a) Hematoxylin and eosin cross-sectional area (CSA) analysis of transversal sections of the indicated muscles of 90-day-old AR113Q and CTR (wild type) mice revealed that the CSA of these glycolytic muscles is decreased in AR113Q compared to CTR mice. Graphs show mean ± sem, n = 3 mice, n fibers = 2,125 CTR, 2,377 AR113Q (quadriceps); 1,398 CTR, 1,975 AR113Q (gastrocnemius); 1,263 CTR, 1,579 AR113Q (tibialis anterior).
b) Hematoxylin and eosin CSA analysis of transversal sections of the indicated muscles of 90-day-old AR113Q and AR21Q mice confirmed that the CSA of quadriceps, gastrocnemius, and TA muscles is decreased in AR113Q mice compared to AR21Q mice. Graphs show mean ± sem, n = 3 mice, n fibers = 1,836 AR21Q, 2,377 AR113Q (quadriceps); 1,706 AR21Q, 1,975 AR113Q (gastrocnemius); 1,059 AR21Q, 1,579 AR113Q (TA).
c) Hematoxylin and eosin CSA analysis of transversal sections of the indicated muscles of 180-day-old AR113Q and CTR (wild type) mice showed that the CSA of soleus is increased, whereas that of gastrocnemius is decreased in AR113Q mice. Graphs show mean ± sem, n = 3 mice, n fibers = 2,356 CTR, 2,409 AR113Q (gastrocnemius); 3,322 CTR, 2,282 AR113Q (soleus).

Supplementary Figure 3

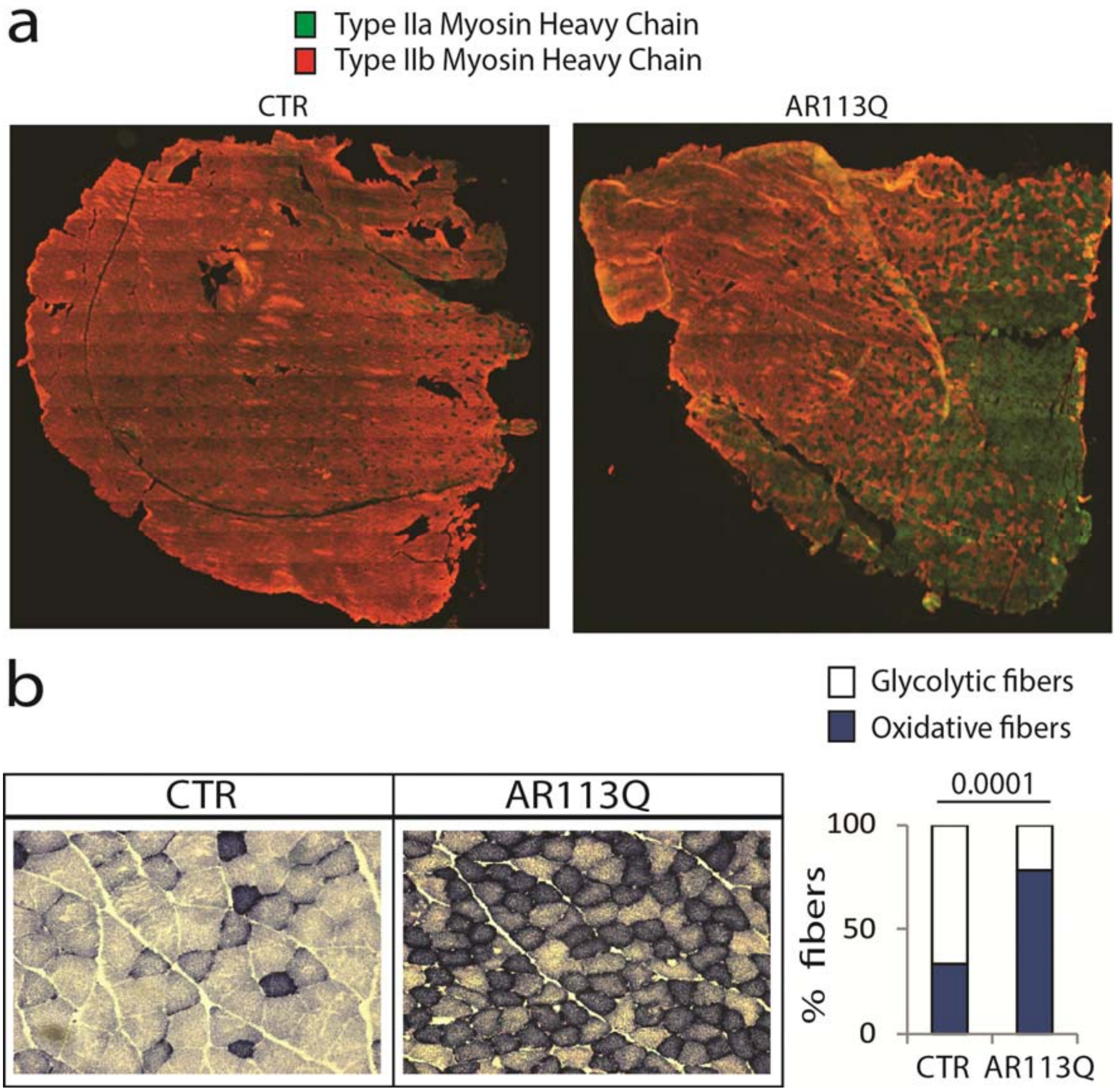

Supplementary Figure 3. Glycolytic-to-oxidative fiber-type switch in the quadriceps and gastrocnemius muscles of AR113Q mice.

a) Immunofluorescence analysis of type IIa (green) and IIb (red) myosin heavy chain-positive fibers in the quadriceps muscle of 180-day-old AR113Q and CTR (wild type) mice showed that the number of type IIa fibers is increased in SBMA muscle. Shown are representative images of quadriceps muscle transversal sections (n = 3 mice).

b) NADH staining of transversal sections of gastrocnemius muscle from 180-day-old AR113Q and CTR (wild type) mice revealed that the number of oxidative fibers is increased in AR113Q mice. Graph shows mean ± sem, n mice = 3-5, n fibers = 1,298 AR113Q and 1,273 CTR.

Supplementary Figure 4

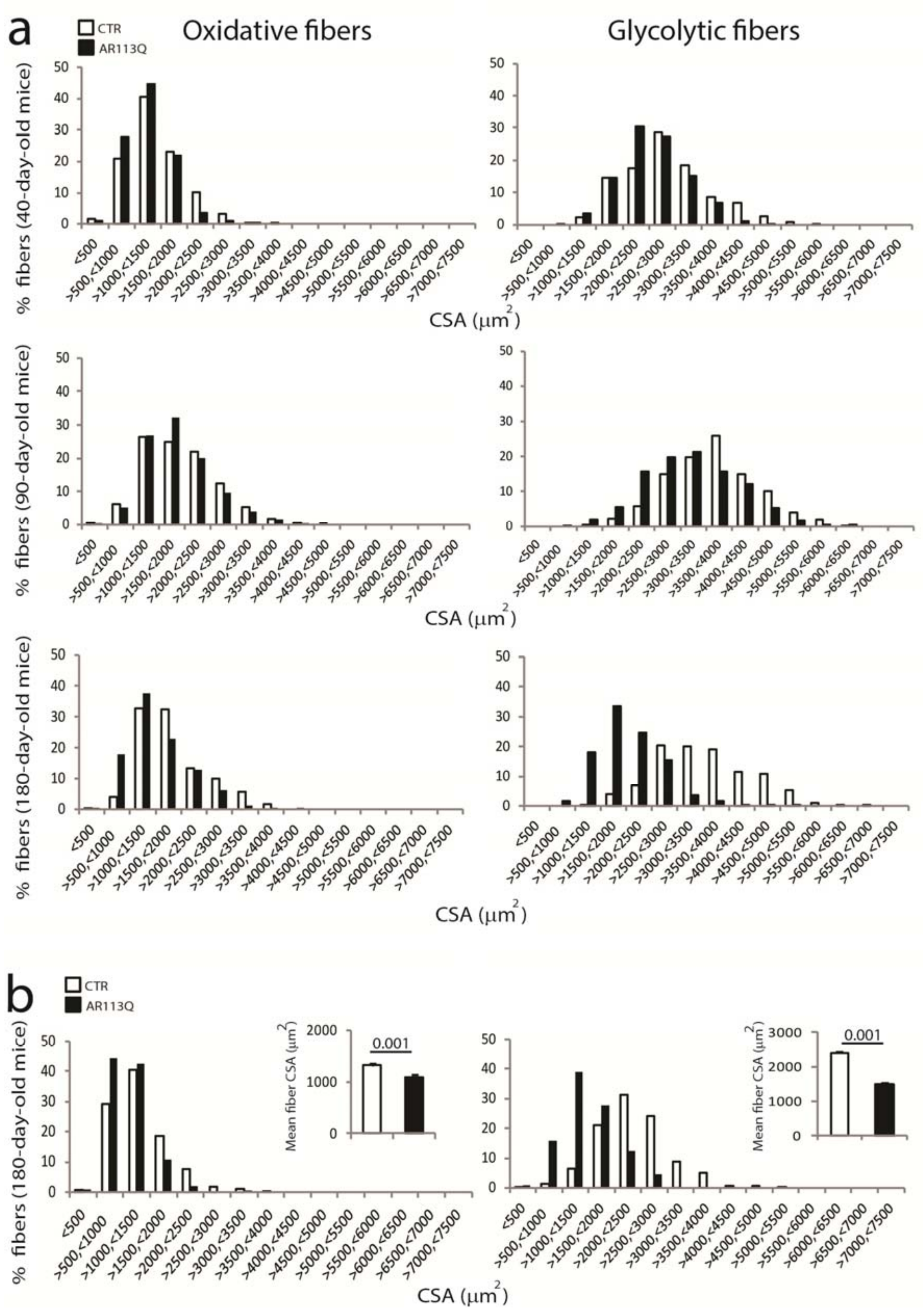

Supplementary Figure 4. Atrophy of glycolytic fibers exceeds that of oxidative fibers in AR113Q mice.

a) CSA distribution of oxidative and glycolytic fibers of quadriceps in 40-, 90-, and 180-day-old AR113Q and CTR (wild type) mice. Graphs show mean ± sem; 40-day-old mice: n mice = 5 AR113Q and CTR, n fibers = 1,490 AR113Q and 1,129 CTR; 90-day-old mice: n mice = 8 AR113Q and CTR, n fibers = 1,674 AR113Q and 1,401 CTR; 180-day-old mice: n mice = 3 AR113Q and 5 CTR, n fibers = 880 AR113Q and 771 CTR.

b) CSA distribution of oxidative and glycolytic fibers of gastrocnemius in 180-day-old AR113Q and CTR (wild type) mice. Graphs show mean ± sem, n mice = 3-5 for each group, n fibers = 1,298 AR113Q and 1,273 CTR.

Supplementary Figure 5

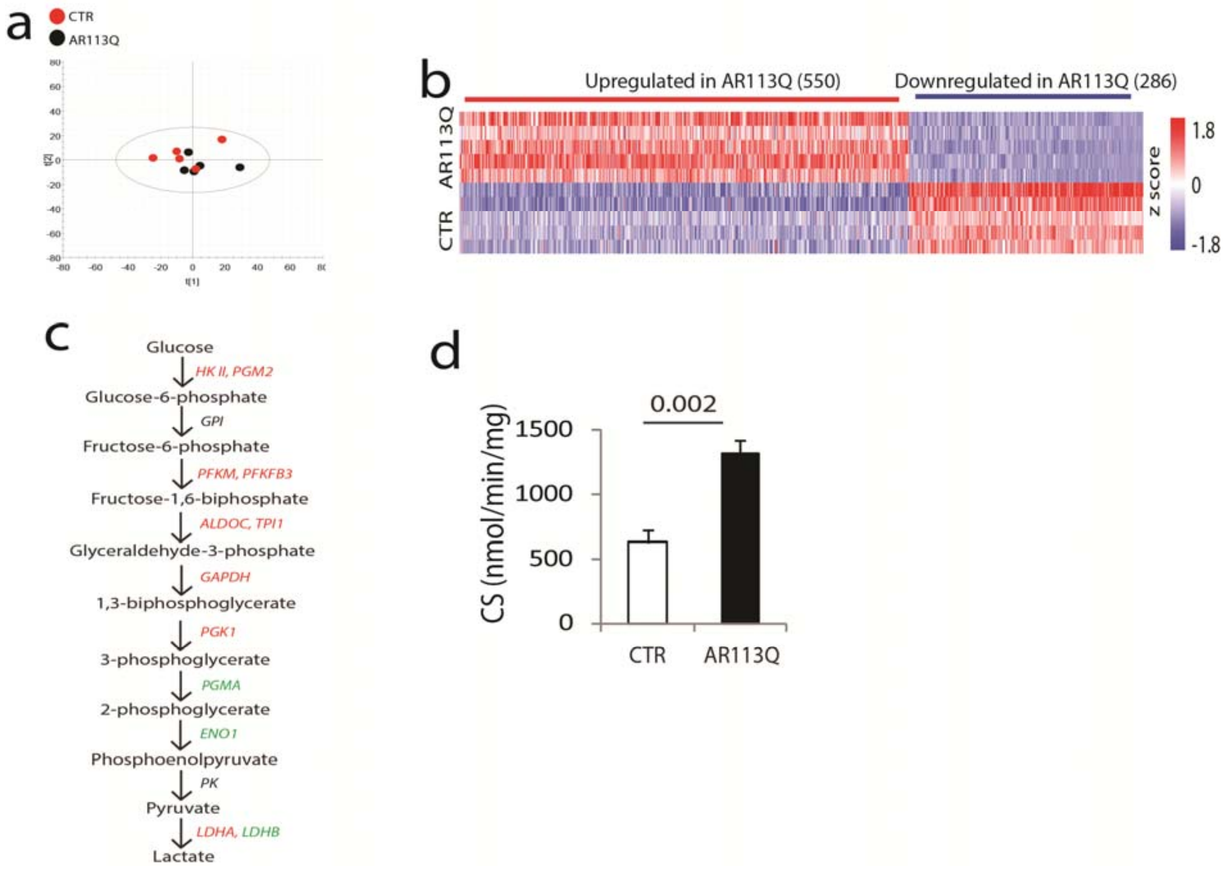

Supplementary Figure 5. Lipidomic and microarray analyses in AR113Q and control mice.

a) Score plot from principal component analysis of high-resolution LC-MS/MS shotgun lipidomic analysis in the serum of 180-day-old AR113Q and CTR (wild type) mice revealed that the serum lipid composition in AR113Q mice is similar to that of CTR mice (n = 6-7 mice).
b) Microarray analysis in the quadriceps muscle of 180-day-old AR113Q and CTR (wild type) mice (n = 5 mice).
c) Microarray analysis revealed altered expression of the transcript levels of glycolytic genes. Red, downregulated genes; green, upregulated genes; black, unchanged genes.
d) Enzymatic activity of citrate synthase (CS) in the quadriceps of 180-day-old AR113Q and CTR (wild type) mice. Graph shows mean ± sem, n = 3-5 mice.

Supplementary Figure 6

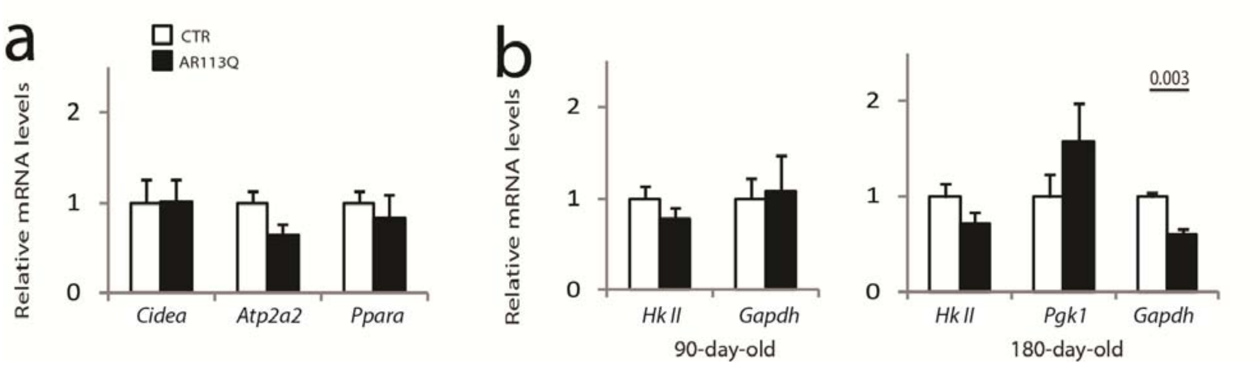

Supplementary Figure 6. The transcript levels of lipid and glycolytic genes in the soleus muscle of AR113Q mice.

a) Real-time PCR analysis of the transcript levels of lipid genes normalized to actin in the soleus muscle of 180-day-old AR113Q and CTR (wild type) mice revealed that the transcript levels of these lipid genes are not increased in the soleus muscle of AR113Q mice. Graph shows mean ± sem, n = 3-4 mice.

b) Real-time PCR analysis of the transcript levels of glycolytic genes normalized to actin in the soleus muscle of 90- and 180-day-old AR113Q and CTR (wild type) mice revealed that *Gapdh*, but not *HkII* and *Pgk1*, transcript levels are downregulated in the soleus of 180-day-old AR113Q mice. Graphs show mean ± sem, n = 3-4 mice.

Supplementary Figure 7

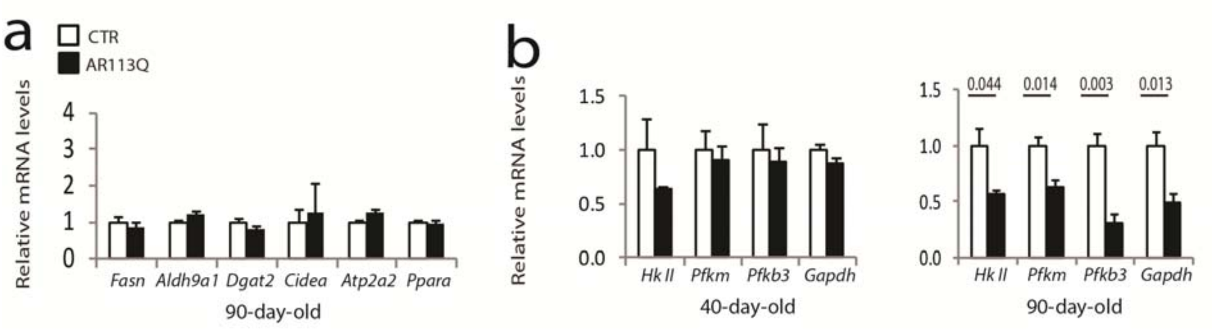

Supplementary Figure 7. Lipid and glycolytic gene expression alterations occur at different ages in the quadriceps muscle of AR113Q mice.

a) Real-time PCR analysis of the transcript levels of lipid genes normalized to beta-glucuronidase in the quadriceps muscle of AR113Q and CTR (wild type) mice showed that at 90 days of age lipid gene expression is not altered in the muscle of AR113Q mice. Graph shows mean ± sem, n = 5 mice.
b) Real-time PCR analysis of the transcript levels of glycolytic genes normalized to beta-glucuronidase in the quadriceps muscle of AR113Q and CTR (wild type) mice showed that at 40 days of age glycolytic gene expression is similar in AR113Q and CTR mice, whereas it is downregulated at 90 days of age. Graph shows mean ± sem, n = 5 mice.

Supplementary Figure 8

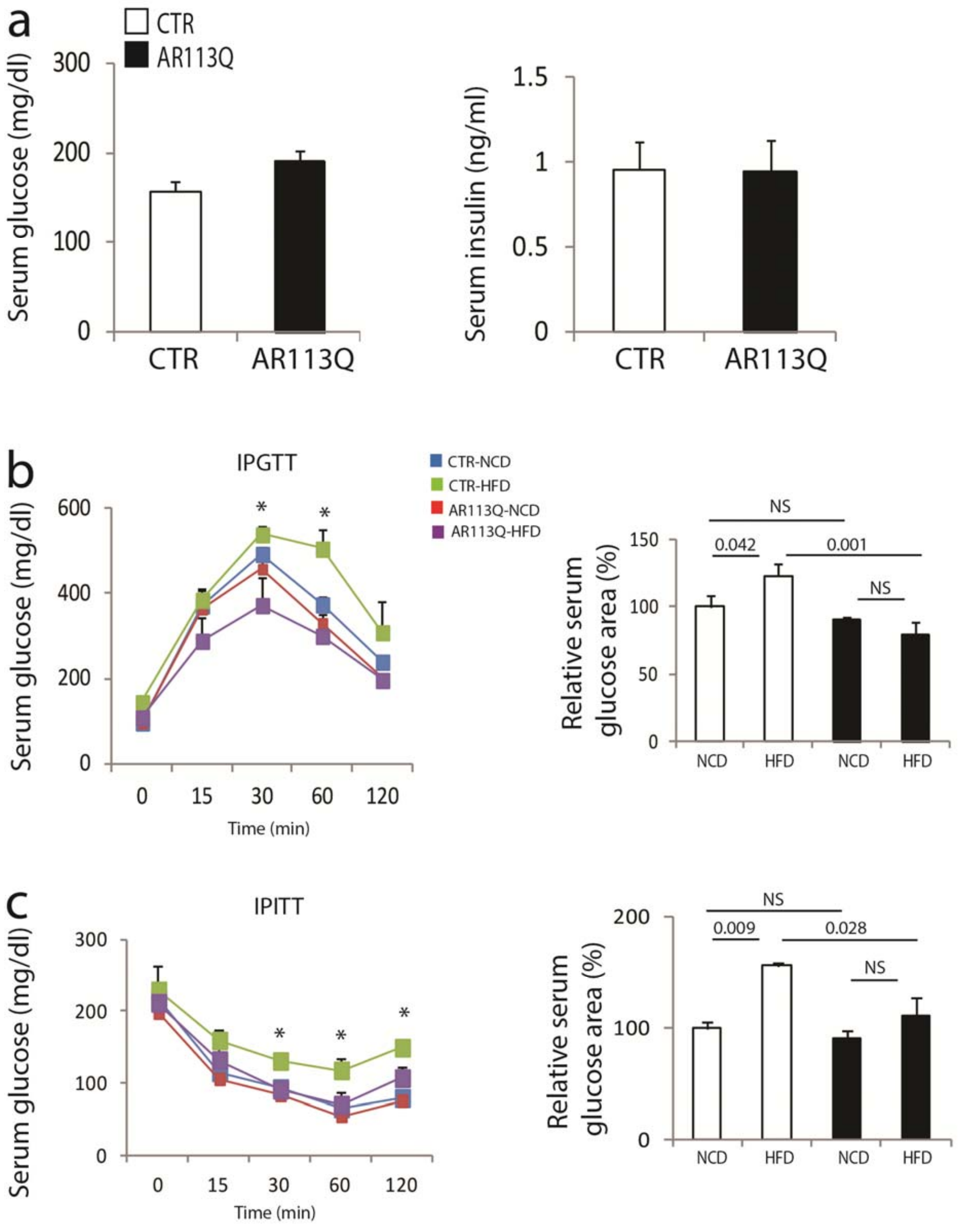

Supplementary Figure 8. Glucose and insulin serum levels in AR113Q and control mice fed either the normal chow diet or the high-fat diet.

a) Steady-state serum glucose and insulin levels were measured upon 4 hour fasting in AR113Q and CTR (wild type) mice fed a normal chow diet. Steady-state glucose and insulin levels were normal in AR113Q mice. Graphs, mean ± sem, n = 9-11 mice.

b) Intraperitoneal glucose tolerance test (IPGTT) performed in 180-day-old AR113Q and CTR (wild type) mice fed either a normal chow diet (NCD) or a high-fat diet (HFD) upon 18 hour fasting. IPGTT revealed glucose intolerance in CTR-HFD mice, but not AR113Q-HFD mice. Graphs show mean ± sem, n = 4 mice, *0.05.

c) Intraperitoneal insulin tolerance test (IPITT) performed in 180-day-old AR113Q and CTR (wild type) mice fed either a normal chow diet (NCD) or a high-fat diet (HFD) upon 4 hour fasting. IPITT revealed insulin resistance in CTR-HFD mice, but not AR113Q-HFD mice. Graphs, mean ± sem, n = 4 mice, *0.05.

Supplementary Figure 9

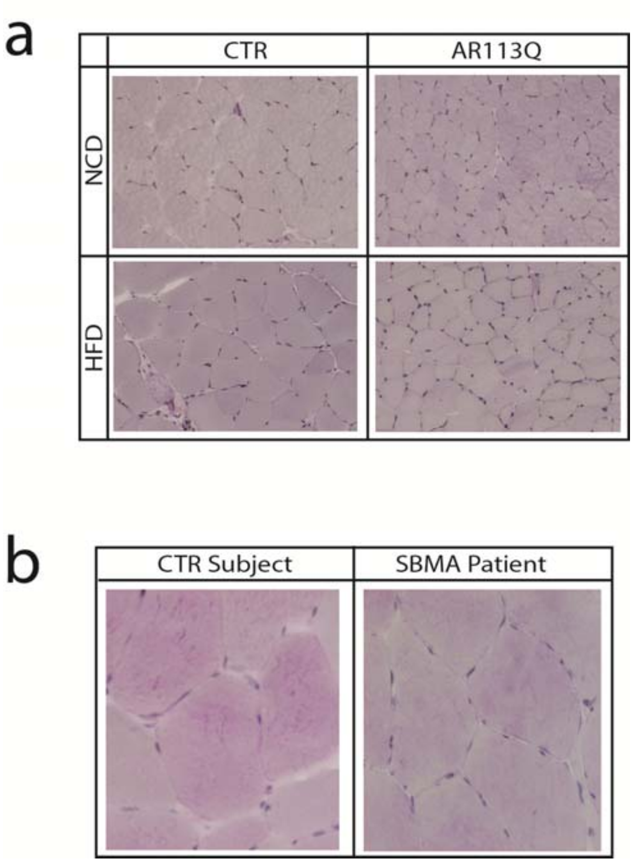

Supplementary Figure 9. Similar glycogen storage in the quadriceps muscle of AR113Q and control mice and SBMA patients.

a) Periodic Acid-Schiff (PAS) staining of transversal sections of the quadriceps muscle of 180-day-old AR113Q and CTR (wild type) mice fed either a normal chow diet (NCD) or a high-fat diet (HFD) revealed that glycogen storage is not altered in the muscle of SBMA knock-in mice. Shown are representative images of n = 3 mice.

b) PAS staining in the transversal sections of quadriceps muscle biopsy specimens derived from CTR subjects and SBMA patients revealed that glycogen storage in muscle is not altered in SBMA patients. Shown are representative images of n = 18 subjects.

Supplementary Figure 10

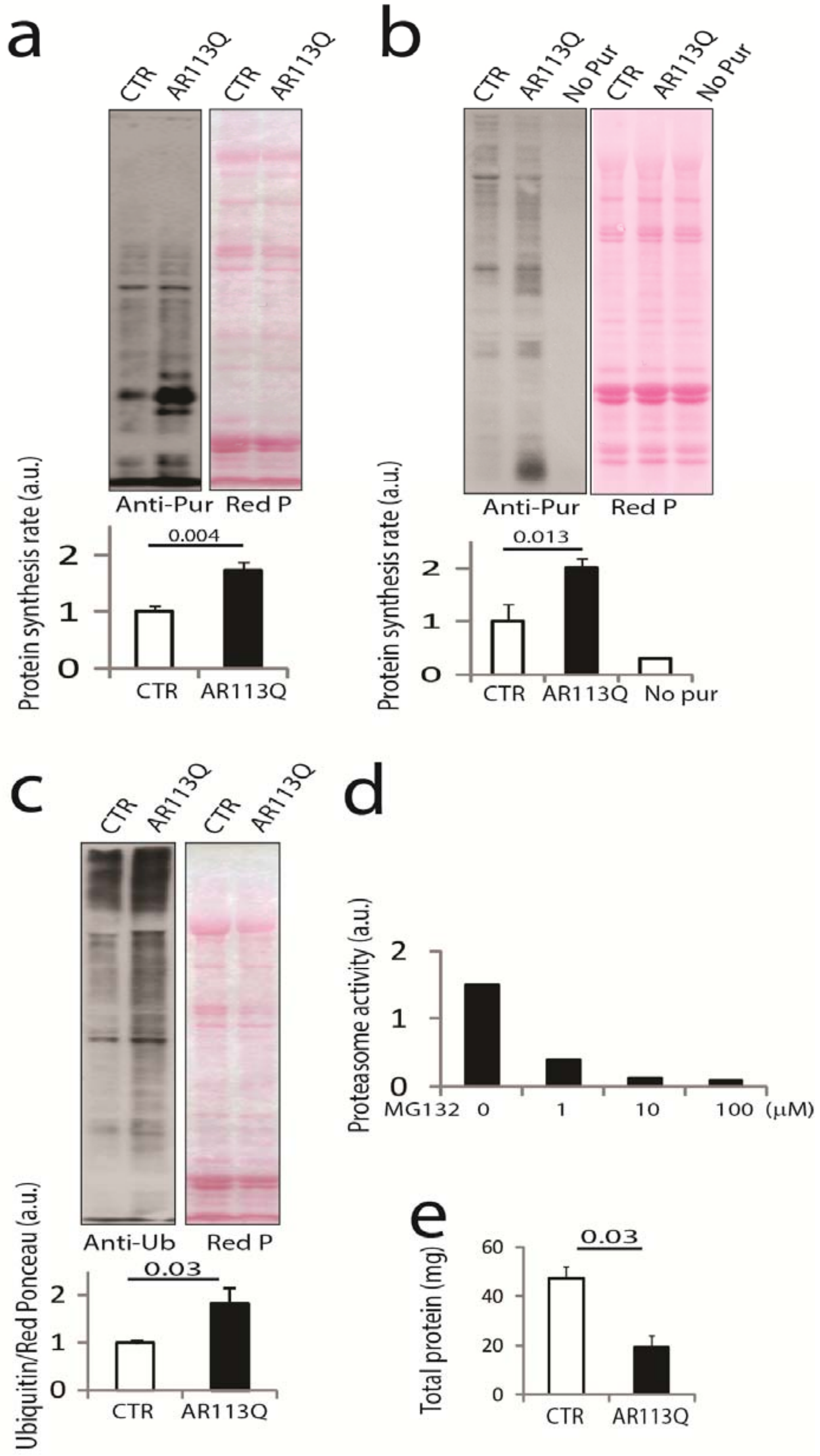

Supplementary Figure 10. Sustained enhancement of protein turnover in SBMA muscle.

a) Western blotting analysis of the rate of new protein synthesis in the quadriceps of 270-day-old AR113Q and CTR (wild type) mice showed that protein synthesis is increased at late stages of disease in AR113Q mice. Puromycin incorporation was detected with anti-puromycin (anti-Pur) antibody, and Red Ponceau (Red P) was used as loading control. Graph shows mean ± sem, n = 4-5 mice.

b) Western blotting as in (a) in mice upon 4 hours fasting showed that protein synthesis is increased in AR113Q mice even upon fasting. Graph shows mean ± sem, n = 6-8 mice.

c) Western blotting analysis of protein ubiquitination in the quadriceps of 270-day-old mice showed that protein degradation is increased in AR113Q mice. Ubiquitinated proteins were detected with anti-ubiquitin (anti-Ub) antibody. Graph shows mean ± sem, n = 4-5 mice.

d) Proteasome activity was measured in muscle lysates in the presence of the indicated concentrations of the proteasome inhibitor, MG132. As expected, MG132 inhibited proteasome activity in a dose-dependent manner, indicating that the signal measured in tissues is specific.

e) Total protein content was decreased in the quadriceps of 270-day-old AR113Q mice compared to CTR mice. Graph shows mean ± sem, n = 4-5 mice.

Supplementary Figure 11

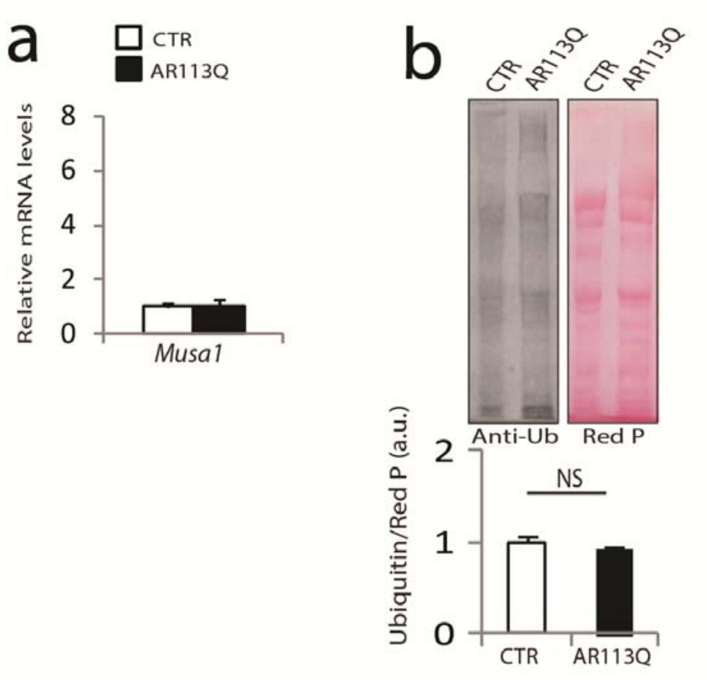

Supplementary Figure 11. Proteasome-mediated protein degradation is not enhanced in the oxidative muscles of AR113Q mice.

a) Real-time PCR analysis of *Musa1* mRNA transcript levels normalized to actin in the soleus muscle of 180-day-old AR113Q and CTR (wild type) mice revealed that this E3 ubiquitin ligase is not induced in the soleus muscle of AR113Q mice. Graph shows mean ± sem, n = 3-4 mice.

b) Western blotting analysis of protein ubiquitination in the soleus of 180-day-old AR113Q and CTR (wild type) mice revealed that the level of protein ubiquitination is not increased in the soleus muscle of AR113Q mice. Ubiquitinated proteins were detected with anti-ubiquitin (anti-Ub) antibody, and Red Ponceau (Red P) was used as loading control. Graph shows mean ± sem, n = 4 mice.

Supplementary Figure 12

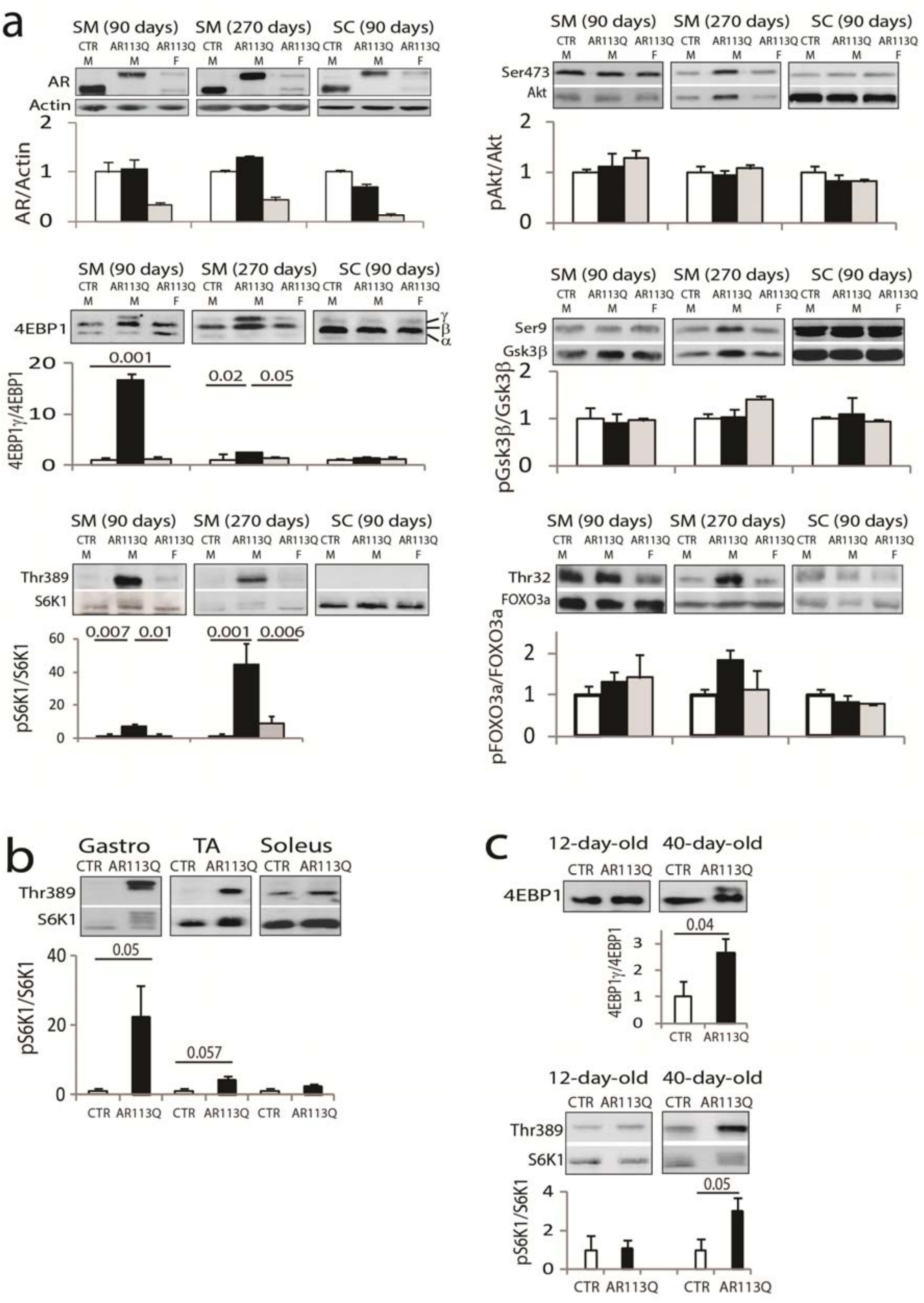

Supplementary Figure 12. Induction of mTOR signaling is not associated with Akt activation and occurs very early specifically in atrophic SBMA muscles.

a) Western blotting analysis of AR expression levels and phosphorylated and total 4EBP1, S6K1 Akt, Gsk3β, and FOXO3a in the quadriceps (SM, skeletal muscle) and spinal cord (SC) of male (M) and female (F) AR113Q mice and CTR (AR21Q) mice. AR expression was similar in male AR113Q and AR21Q mice, and lower in female AR113Q mice. Phosphorylation of 4EBP1 and S6K1 was enhanced in male AR113Q mice, but not in AR21Q male and AR113Q female mice. Graphs show mean ± sem, n = 3-9 mice.

b) Western blotting analysis of phosphorylated and total S6K1 levels in gastrocnemius (gastro), tibialis anterior (TA), and soleus muscles of 90-day-old AR113Q and CTR (wild type) mice. Graph shows mean ± sem, n = 3 mice.

c) Western blotting analysis of phosphorylated and total 4EBP1 and S6K1 in 12- and 40-day-old AR113Q and CTR (wild type) mice. Graphs show mean ± sem, n = 3 mice.

Supplementary Figure 13

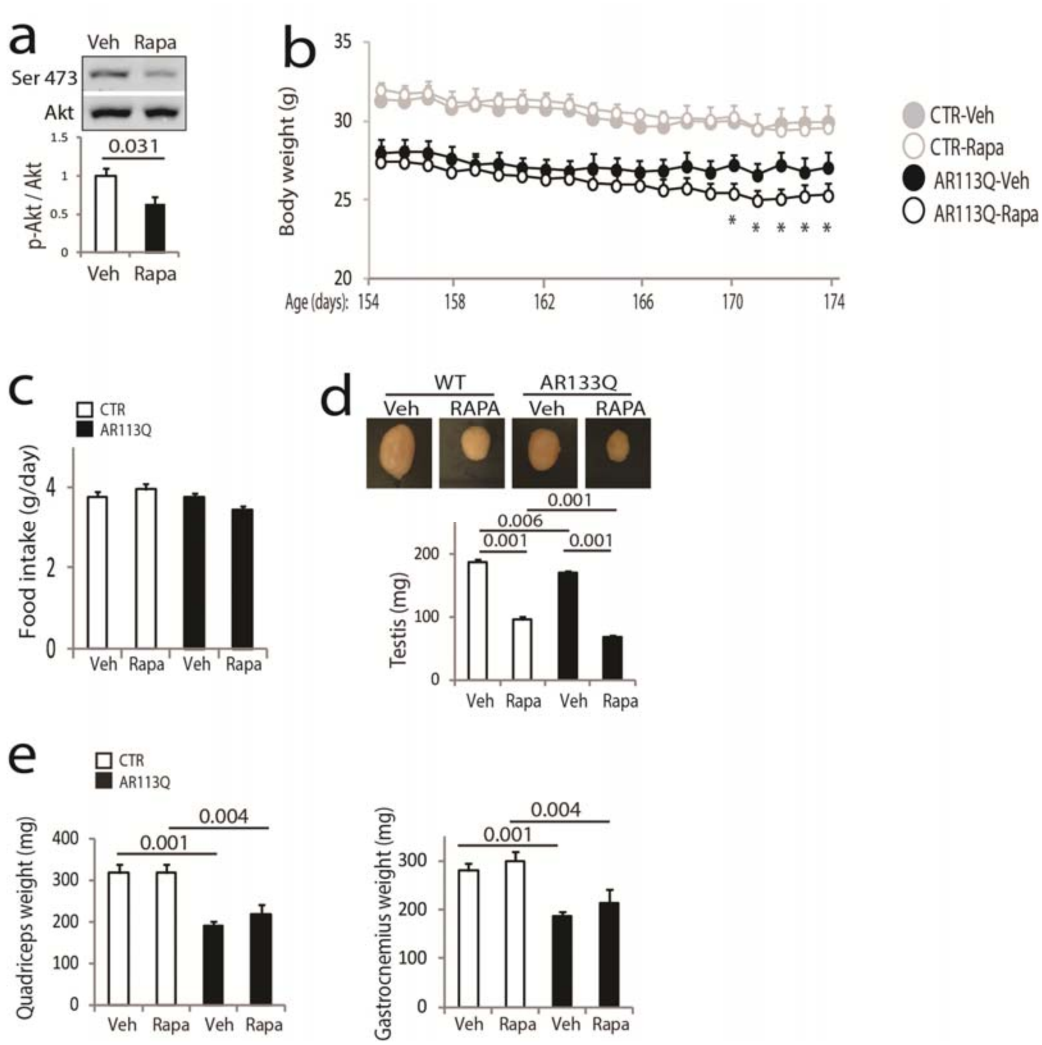

Supplementary Figure 13. Rapamycin treatment results in Akt inhibition and body weight loss in SBMA knock-in mice.

a) Western blotting analysis of Akt phosphorylation in the gastrocnemius of 170-day-old AR113Q mice treated with either vehicle (Veh) or rapamycin (Rapa) revealed that rapamycin treatment decreases the phosphorylation of Akt in glycolytic SBMA muscles. Graph shows mean ± sem, n = 4 mice.
b) Body weight analysis revealed that rapamycin treatment causes body weight loss in AR113Q, but not CTR (wild type), mice. Graph shows mean ± sem, n = 7-11 mice.
c) Food intake analysis showed that rapamycin does not alter the feeding behavior of 170-day-old AR113Q and CTR (wild type) mice. Graph, mean ± sem, n = 5-9 mice.
d) Testis weight in AR113Q and CTR (wild type) mice. Rapamycin caused testicular atrophy in both AR113Q and CTR mice. Graph, mean ± sem, n = 7-11 mice.
e) Quadriceps and gastrocnemius muscle wet weight. Rapamycin did not modify the mass of quadriceps and gastrocnemius muscles of AR113Q mice. Graph, mean ± sem, n = 7-11 mice.

Supplementary Figure 14

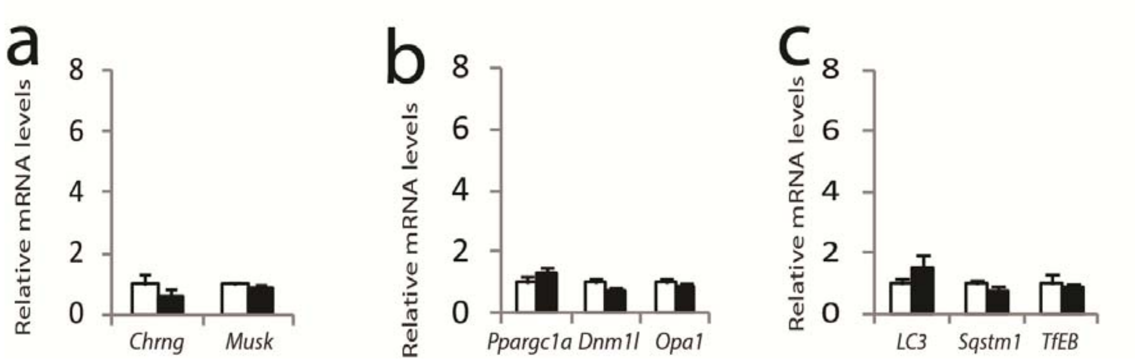

Supplementary Figure 14. Denervation, mitochondrial, and autophagy makers are not altered in the soleus muscle of AR113Q mice.

a-c) Real-time PCR analysis of the transcript levels of denervation markers (a), mitochondrial markers (b), and autophagy markers (c) in the soleus muscle of 180-day-old AR113Q and CTR (wild type) mice revealed that the expression of these genes is not altered by polyglutamine-expanded AR in soleus. Graph, mean ± sem, n = 3-4 mice.

Supplementary Figure 15

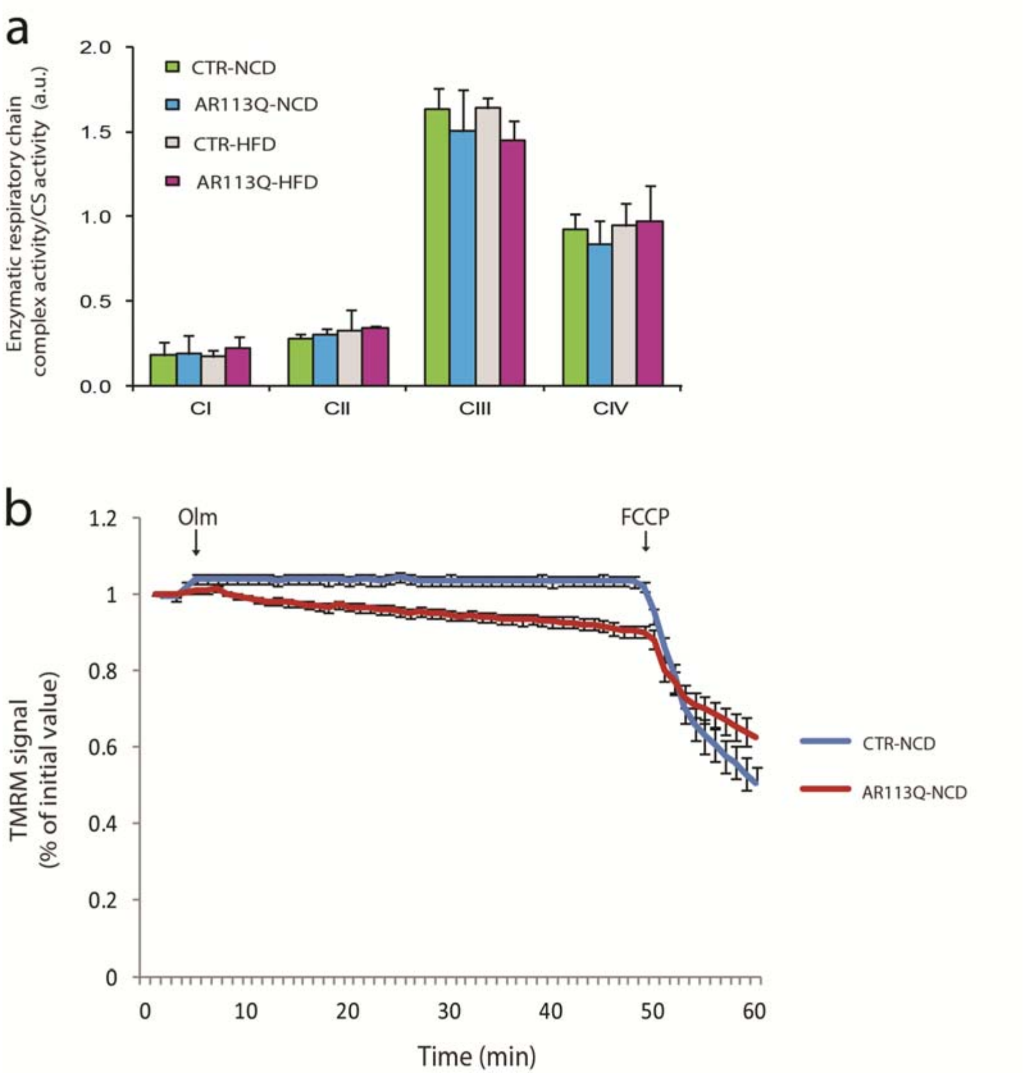

Supplementary Figure 15. Mitochondrial complex activity and membrane depolarization in SBMA knock-in mice.

a) Mitochondrial complex activity measured in the quadriceps of 180-day-old AR113Q and CTR (wild type) mice fed either a normal chow diet (NCD) or a high-fat diet (HFD) and normalized to citrate synthase (CS) activity. Graph shows mean ± sem, n = 3 mice for each group.

b) Mitochondrial membrane depolarization measured in fibers isolated from flexor digitorum brevis of 90-day-old AR113Q and CTR (wild type) mice fed a normal chow diet (NCD). Graph shows mean ± sem, n = 60 fibers from 3 mice for each group. Olm, oligomycin; FCCP, protonophore carbonyl cyanide *p*-trifluoromethoxyphenylhydrazone TMRM, tetramethyl rhodamine methyl ester.

Supplementary Figure 16

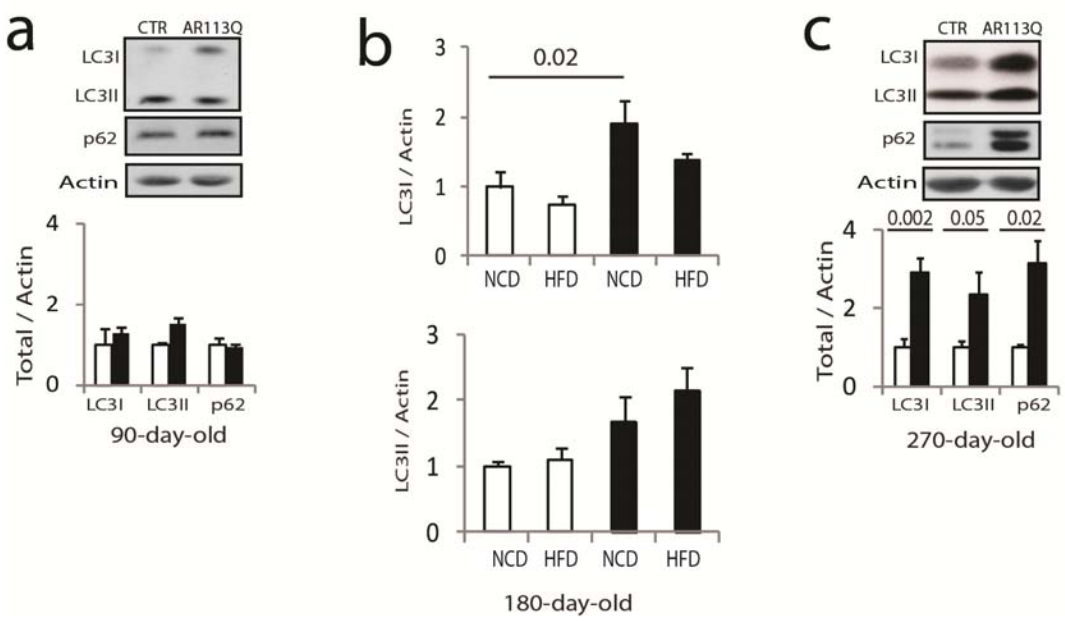

Supplementary Figure 16. Autophagy is induced and altered at late stages of disease in the quadriceps muscle of SBMA knock-in mice.

a-c) Western blotting analysis of LC3I and II and p62 expression levels in the quadriceps of 90-day-old (a), 180-day-old (b), and 270-day-old (c) AR113Q and CTR (wild type) mice. The levels of expression and post-translational modifications of LC3 and p62 were similar in AR113Q and CTR mice at 90 days of age. LC3I was increased in the muscle of 180-day-old AR113Q mice. LC3I, LC3II, and p62 accumulated in the muscle of 270-day-old AR113Q mice, suggesting a block of autophagy flux. Graphs show mean ± sem, n = 3-4 mice.

.

Supplementary Figure 17

NORMAL CHOW

Formula

Product #D12450B	gm%	kcal%
Protein	19.2	20
Carbohydrate	67.3	70
Fat	4.3	10
Total		100
kcal/gm	3.85	

Ingredient	gm	kcal
Casein, 30 Mesh	200	800
L-Cystine	3	12
Corn Starch	315	1260
Maltodextrin 10	35	140
Sucrose	350	1400
Cellulose, BW200	50	0
Soybean Oil	25	225
Lard*	20	180
Mineral Mix S10026	10	0
DiCalcium Phosphate	13	0
Calcium Carbonate	5.5	0
Potassium Citrate, 1 H2O	16.5	0
Vitamin Mix V10001	10	40
Choline Bitartrate	2	0
FD&C Yellow Dye #5	0.05	0
Total	1055.05	4057

HIGH-FAT CHOW

Formula

Product #D12451	gm%	kcal%
Protein	24	20
Carbohydrate	41	35
Fat	24	45
Total		100
kcal/gm	4.73	

Ingredient	gm	kcal
Casein, 30 Mesh	200	800
L-Cystine	3	12
Corn Starch	72.8	291
Maltodextrin 10	100	400
Sucrose	172.8	691
Cellulose, BW200	50	0
Soybean Oil	25	225
Lard*	177.5	1598
Mineral Mix S10026	10	0
DiCalcium Phosphate	13	0
Calcium Carbonate	5.5	0
Potassium Citrate, 1 H2O	16.5	0
Vitamin Mix V10001	10	40
Choline Bitartrate	2	0
FD&C Red Dye #40	0.05	0
Total	858.15	4057

Supplementary Figure 17. Diet formula.

Supplementary Figure 18

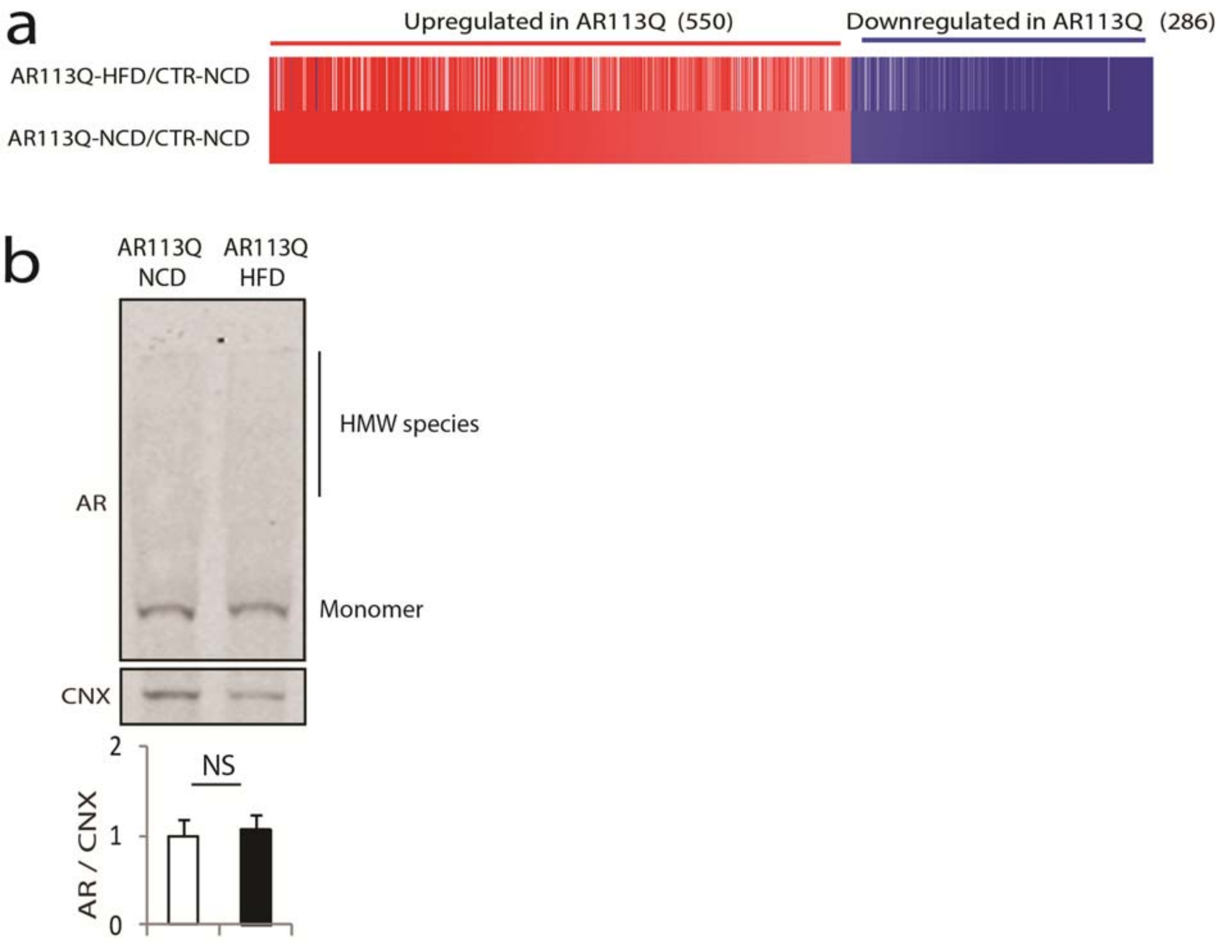

Supplementary Figure 18. The effect of high-fat diet on gene transcription and polyglutamine-expanded AR expression levels in the quadriceps muscle of AR113Q mice.

a) Microarray analysis in the muscle of 180-day-old AR113Q and CTR (wild type) mice fed the HFD compared to animals fed the NCD.

b) Western blotting analysis of AR expression levels in the quadriceps muscle of 180-day-old AR113Q mice fed either a normal chow diet (NCD) or a high-fat diet (HFD) revealed that the HFD does not affect the levels of expression of polyglutamine-expanded AR. Notice that no accumulation of high molecular weight (HMW) species was detected in protein lysates containing 2% SDS, indicating that in these mice polyglutamine-expanded AR does not form 2% SDS-resistant aggregates. Graph shows mean ± sem, n = 3 mice.

Supplementary Figure 19

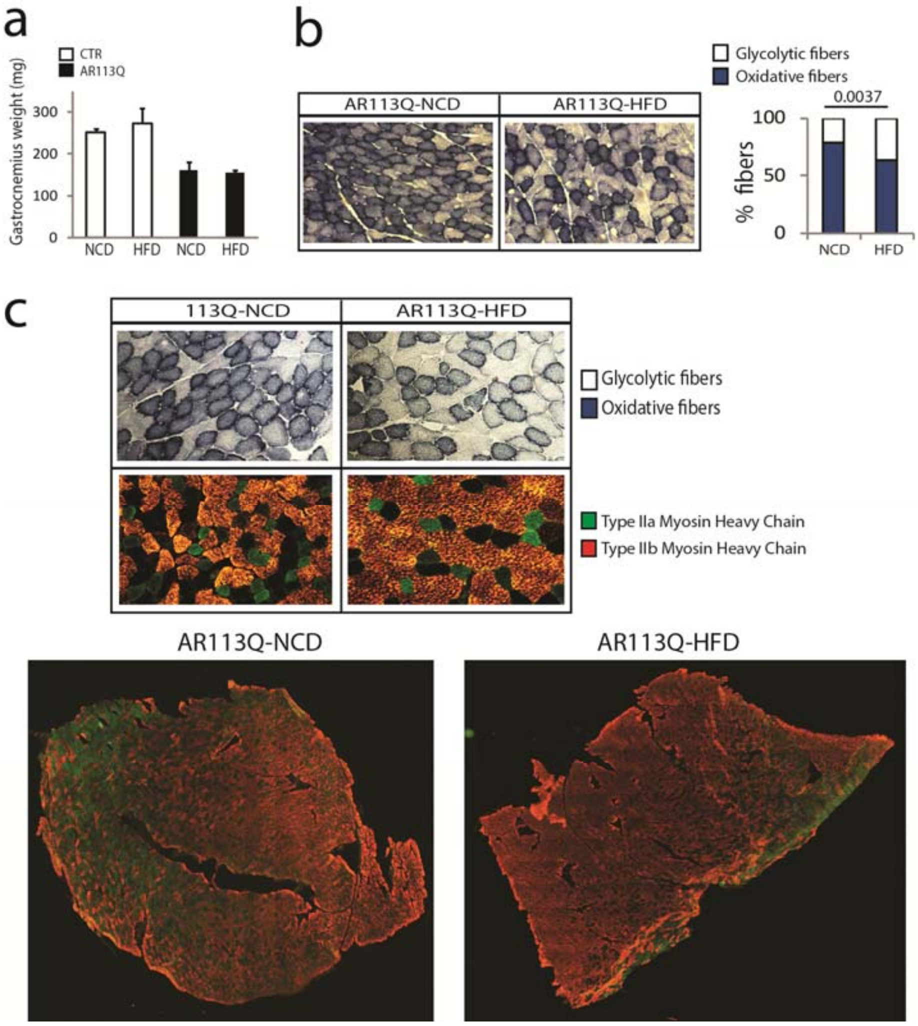

Supplementary Figure 19. The high-fat diet attenuates the glycolytic-to-oxidative fiber-type shift in the quadriceps and gastrocnemius muscles of AR113Q mice.

a) Wet weight of gastrocnemius muscle in AR113Q and CTR (wild type) mice fed either the normal chow diet (NCD) or the high-fat diet (HFD). The HFD did not modify the mass of gastrocnemius of 180-day-old AR113Q mice. Graph, mean ± sem, n = 4-8 mice.

b) NADH staining of gastrocnemius from 180-day-old AR113Q mice fed either the NCD or HFD revealed that the HFD reduces the number of oxidative fibers in the gastrocnemius of AR113Q mice. Graphs, mean ± sem, n mice = 3 NCD-fed and 4 HFD-fed, n fibers = 1298 AR113Q-NCD, 1440 AR113Q-HFD.

c) NADH and immunofluorescence analyses of type IIa (green) and IIb (red) myosin heavy chain-positive fibers in the quadriceps muscle of 180-day-old AR113Q-NCD and AR113Q-HFD mice. Shown are representative images of selected areas (top panels) and entire (bottom panels) muscle transversal sections (n = 3 mice).

Supplementary Figure 20

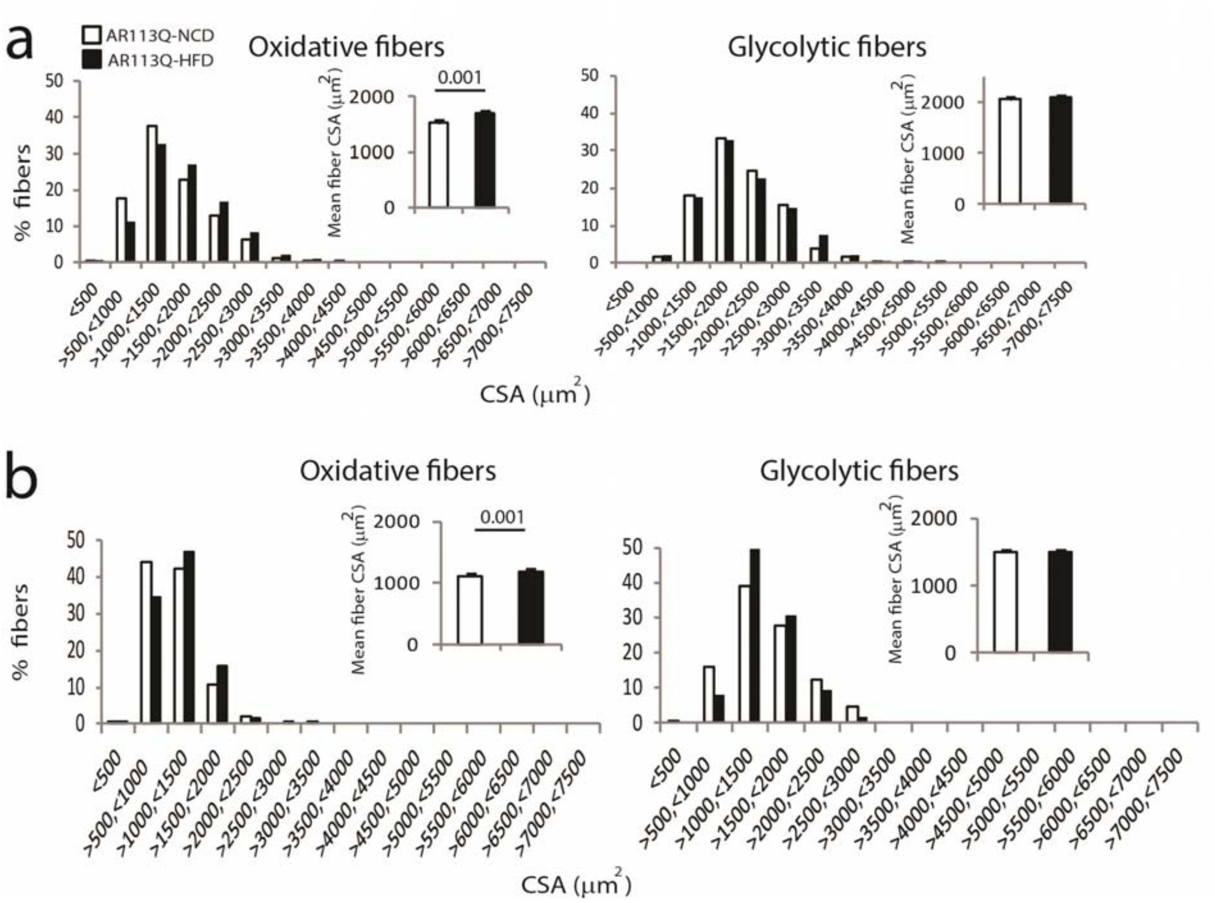

Supplementary Figure 20. The high-fat diet increases the CSA of oxidative fibers in AR113Q mice.

a) Analysis of the CSA distribution of oxidative and glycolytic fibers in the quadriceps muscle of 180-day-old AR113Q-NCD and AR113Q-HFD mice revealed that the HFD increases the CSA of oxidative, but not glycolytic fibers. Graph, mean ± sem, n mice = 3 AR113Q-NCD and 4 AR113Q-HFD, n fibers = 880 AR113Q-NCD and 1,017 AR113Q-HFD.

b) Analysis of the CSA distribution of oxidative and glycolytic fibers in the gastrocnemius muscle of 180-day-old AR113Q-NCD and AR113Q-HFD mice. Graphs, mean ± sem, n mice = 3 NCD-fed and 4 HFD-fed, n fibers = 1298 AR113Q-NCD, 1440 AR113Q-HFD.

Supplementary Figure 21

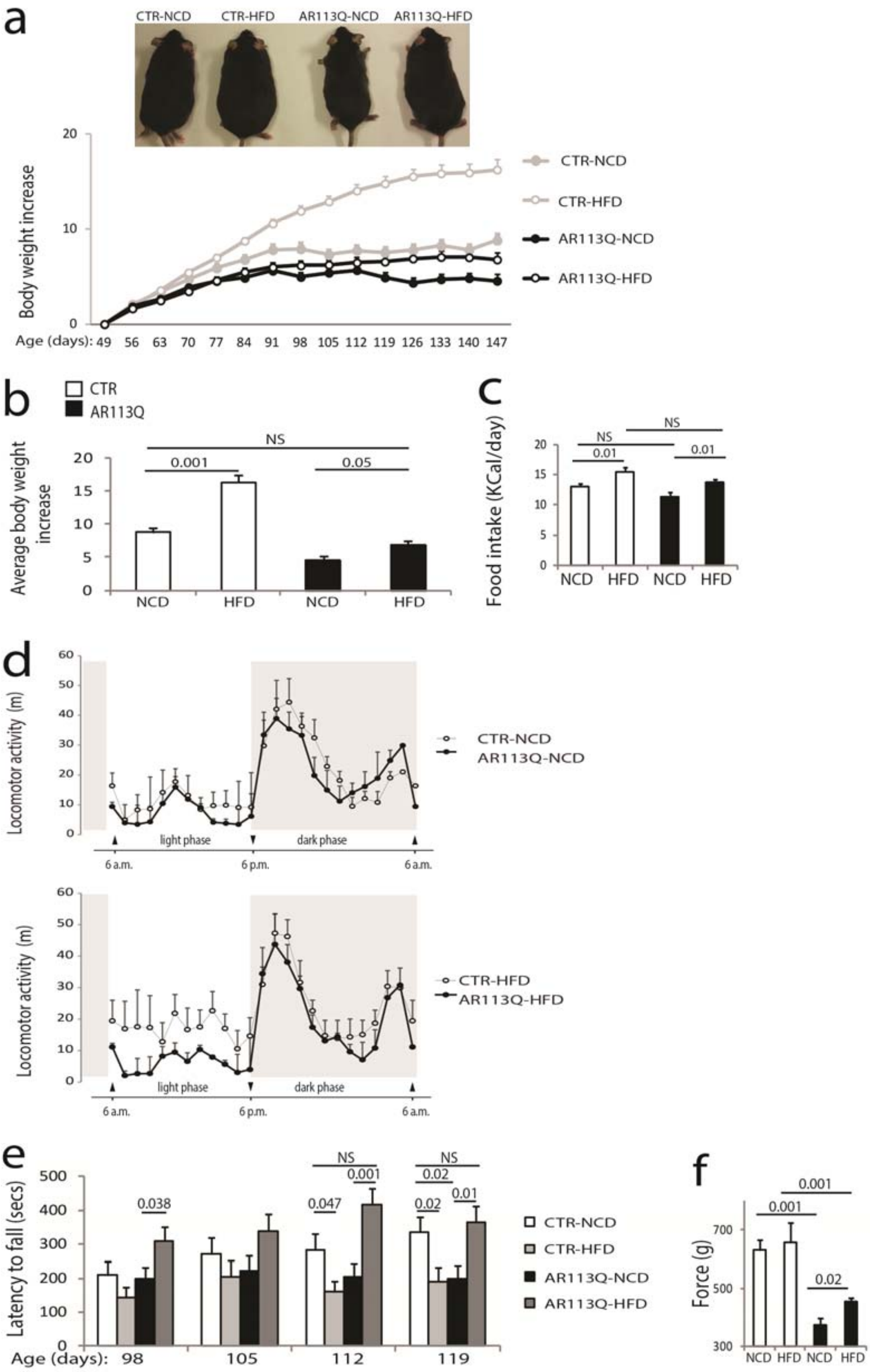

Supplementary Figure 21. Effect of the high-fat diet on the phenotype of AR113Q and CTR mice.

a) Body weight increase in the AR113Q and CTR (wild type) mice fed the normal chow diet (NCD) and the high-fat diet (HFD). Graph, mean ± sem, n = 18-21 mice.
b) Average body weight increase. Graph, mean ± sem, n = 18-21 mice.
c) Food intake. Graph, mean ± sem, n = 5-6 mice.
d) Locomotor activity in AR113Q and CTR mice fed the NCD (upper panel) and HFD (bottom panel). Locomotor activity was recorded by a video tracking system with computer interface and video camera (ANY-maze; Stoelting). Parameters were set within the ANY-maze program to ensure continuous movement tracking. Distance traveled within the cage was analyzed as a measure of activity. Mouse locomotor activity was assessed every hour for 48 hours. Graph, mean ± sem, n = 5-8 mice.
e) Rotarod analysis. Graph shows mean ± sem, n = 20-25 mice.
f) Force of gastrocnemius muscle measured in living 180-day-old AR113Q and CTR (wild type) mice. Graph shows mean ± sem, n = 8.

Materials and methods

Animals and treatments

Animal care and experimental procedures were conducted in accordance with the Italian Institute of Technology and the University of Trento ethical committees and were approved by the Italian Ministry of Health. Generation and genotyping of knock-in AR21Q and AR113Q mice were previously described [5]. Male mice were used in this study, and AR113Q female mice were used as indicated (**Supplementary Figure 12**). Mice were housed in filtered cages in a temperature-controlled room with a 12-hour light/12-hour dark cycle with *ad libitum* access to water and food. Mice were fed a standard diet (Mucedola 4RF21), purified normal chow diet (NCD, D12450B Research Diet), and high-fat diet (HFD, D12451 Research Diet) as indicated. Forty-day-old mice were randomly assigned to NCD and HFD groups and kept in diet regime until the end of the study. The operator was blind for genotype and treatment. Locomotor activity was recorded by a video tracking system with computer interface and video camera (ANY-maze; Stoelting). Parameters were set within the ANY-maze program to ensure continuous movement tracking. Distance traveled within the cage was analyzed as a measure of activity. Mouse locomotor activity was assessed every hour for 48 hours. For rotarod analysis (Ugo Basile), mice received a weekly session, which included one trial followed by two test trials at 21 rpm speed for a maximum period of 600 seconds. The average of recordings for each mouse was used. Total distance was quantified as the sum of the distance measured (in centimeters) during all tests. For rapamycin treatment, 154-day-old mice were injected intraperitoneally with rapamycin (4.0 mg/kg, Gold Biotechnology). Rapamycin was reconstituted in ethanol at 20 mg/ml and then diluted to a final concentration in 5% polyethylene glycol 400 (Sigma) and 5% Tween 80 (Sigma) immediately prior to injection. Rapamycin and vehicle-treated mice received the same volume of ethanol. Mice were sacrificed 12 hours after the last injection. Body weight and food intake were measured weekly from week 7 through week 21 of age. For analysis of the rate of protein synthesis, animals were starved 30 minutes, injected with puromycin (0.040 µmol/g puromycin dissolved in 100 µl PBS), and sacrificed 30 minutes after injection. Tissues were collected and processed for Western blotting analysis.

Human samples

Anonymized control and patient biopsy sample collection was approved by the ethics committee of the University of Padova (Italy). Written informed consent was obtained from each patient. Confidentiality was guaranteed by assigning a study code to each patient. All patients who underwent muscle biopsy were clinically affected and showed weakness and/or fasciculation and/or muscle atrophy. Myopathic changes together with neurogenic atrophy were observed in muscle biopsies.

Histological analysis

Muscles were collected immediately after euthanasia, flash-frozen in isopentane precooled in liquid nitrogen, and stored at -80°C until further processing. Frozen muscles were embedded in optimal cutting temperature (OCT) compound (Tissue Tek, Sakura), and cross-sections (10 µm thick) were cut with a cryostat (CM1850 UV, Leica Microsystems). Cryosections of muscles were processed for hematoxylin and eosin (H&E) and

nicotinamide adenine dinucleotide (NADH) staining. For H&E staining, sections were air-dried and incubated in hematoxylin (Sigma) for 3 minutes, then washed in water and incubated in eosin (Roth) for 1 minute. Sections were then washed in water and dehydrated rapidly in 70%, 80%, and 100% ethanol, and xylene. Sections were mounted with di-N-butyl phthalate in xylene (DPX) mounting media (Sigma). For NADH, sections were air-dried and incubated in NADH solution [0.2M TRIS-HCL, Nitrotetrazolium Blue chloride (Sigma), β-Nicotinamide adenine dinucleotide, (Sigma)] at 37°C for 40 minutes. Sections were briefly washed in 30%, 60%, 90%, 60%, and 30% acetone solutions. Sections were then washed in water, dried, and mounted with aqueous mounting medium [Gelatin (Serva), sodium azide (Merck), and glycerin]. Images were taken using a Nikon Eclipse 90i upright microscope, the analyses were performed using ImageJ, and pixel number was converted to μm^2. The number of fibers and mean cross-sectional area (CSA) distribution were evaluated by a blinded investigator from 3-5 randomly selected areas of 3 sections of each muscle for each genotype. For Periodic Acid–Schiff staining (PAS), sections were air-dried and incubated in Carnoy's solution (ethanol, chloroform and glacial acetic acid) for 10 minutes. Then, sections were incubated in 0,5 % periodic acid solution (Merck) for 5 minutes and washed in Schiff's reagent (Merck) for 15 minutes. Sections were washed in water and incubated in hematoxylin (Sigma) for 4 minutes, then washed in water and dehydrated rapidly in 70, 80 and 100 % ethanol, and Xylene. Sections were mounted with di-Nbutyle phthalate in xylene (DPX) mounting media (Sigma). For immunofluorescence analysis, isopentane-frozen muscles were embedded in OCT compound and cross sections (10 µm thick) were cut with a cryostat. Muscle cryosections were incubated with M.O.M. IgG blocking solution (Vector) for 1 hour at room temperature, then briefly washed 3 times with PBS for 5 minutes. Cryosections were incubated for 1 hour at 37°C with a solution of phosphate-buffered saline (PBS) containing 0.5% bovine serum albumin (BSA) and the following monoclonal anti-myosin heavy chain (MyHC) antibodies: BA-D5 (IgG2b, supernatant, 1:100 dilution, Developmental Studies Hybridoma Bank, University of Iowa) specific for MyHC-I; SC-71 (IgG1, supernatant, 1:100 dilution, Developmental Studies Hybridoma Bank, University of Iowa) specific for MyHC-2A; and BF-F3 (IgM, purified antibody, 1:100 dilution, Developmental Studies Hybridoma Bank, University of Iowa) specific for MyHC-2B. Type 2X fibers are not recognized by these antibodies, and therefore appear black. After 3 washes (5 minutes each) with PBS, sections were incubated for 1 hour at room temperature in PBS containing 0.5% BSA and 3 different secondary antibodies (Jackson ImmunoResearch): goat anti-mouse IgG1 conjugated with DyLight488 fluorophore (for SC-71); goat anti-mouse IgG2b conjugated with DyLight405 fluorophore (for BA-D5); goat anti-mouse IgM conjugated with DyLight549 fluorophore (for BF-F3). After 3 washes with PBS (5 minutes each), sections were mounted with aqueous mounting medium (Fluorescence Mounting Medium, Dako). Images were taken using an upright epifluorescence microscope (Zeiss Axio Imager M2) equipped with an X-Cite 120Q fluorescence light source and a Zeiss Mrm Color Camera. Multichannel images and mosaics were taken using Zeiss Axio Vision Software (V.4.8.2 SP3).

Biochemical analysis

Muscles were mechanically pulverized and homogenized in lysis buffer [20 mM HEPES, 5 mM EGTA, 2% sodium dodecyl sulfate (SDS)] containing protease and phosphatase inhibitor cocktails (Roche), boiled for 3

minutes, and centrifuged for 20 minutes at 13,000 rpm at room temperature. The soluble fraction was collected and protein concentration was determined using the bicinchoninic acid (BCA) assay method (Thermo Scientific). For Western blotting, tissue lysates were boiled in 5X sample buffer [62.5 mM Tris-HCl, pH 6.8, 2% SDS, 25% glycerol, 0.05% bromophenol blue, 5% β-mercaptoethanol, deionized water] and resolved by SDS-polyacrylamide gels (SDS-PAGE). Gels were electro-transferred to nitrocellulose membranes (Thermo Scientific; 0.45 μm pore size). The following antibodies were used: Cell Signaling Technology: GAPDH (2118), 4EBP1 (9452S), phospho-Thr389-S6K1 (9205S), total S6K1 (9292), phospho-Ser240/244-S6 (2215), total S6 (2317), phospho-Ser473-Akt (9271S), total Akt (9272), phospho-Ser9-Gsk3β (9323), total Gsk3β (9315), phospho-Thr32-FoxO3a (9664S); Santa Cruz Biotechnology: anti-AR (SC-816), ubiquitin (SC-8017), HKII (SC-6521), total FoxO3a (SC-11351); Sigma: actin (A2066), LC3B (L7543), p62 (P0067); Merck-Millipore: puromycin (Mabe343); Enzo Life Sciences: calnexin (ADI-SPA 860). Signal intensities were quantified by ImageQuant LAS 4000 mini (GE Healthcare BioSciences). For analysis of enzymatic activity, lactate dehydrogenase (LDH) activity was measured in total muscle homogenate by following the rate of NADH oxidation at 340 nm for 3 minutes at 25°C, as previously described [2]. For analysis of enzymatic activity, lactate dehydrogenase (LDH) activity was measured in total muscle homogenate by following the rate of NADH oxidation at 340 nm for 3 minutes at 25°C, as previously described [2]. Citrate synthase activity was assessed in total muscle homogenate by following the rate of 5-thio-2-nitrobenzoic acid (TNB) formation at 412 nm for 5 minutes at 37°C, as previously described [4]. For proteasome activity assay, muscles were lysed using ice-cold lysis buffer [50 mM HEPES, 5 mM EDTA, 150 mM NaCl, 2 mM ATP, 1% TritonX-100], sonicated and centrifuged. Total protein levels were determined using the BCA assay method (Thermo Scientific). Equal amounts (60 μg) of extracts were incubated for 2 hours at 37°C in 70 μl of a solution containing 25 mM HEPES, 0.5 mM EDTA, 0.05% NP40, 0.001% SDS, and 0.5 mM N-Succinyl-Leu-Leu-Val-Tyr-7-Amido-4-Methylcoumarin (Sigma). The absorption was recorded at 460 nm by excitation at 355 nm (Victor3-V luminometer, PerkinElmer). The proteasome inhibitor MG132 (Calbiochem) was used as a control for the assay.

High-resolution LC-MS/MS analysis for lipidomic profiling

Solvents and chemicals were purchased from Sigma and were used without further purification. UPLC/MS and MS/MS systems, columns and data analysis software were from Waters. Homogenized samples were extracted using a Bligh-Dryer extraction: 200 μl of sample was transferred to glass vials. Liquid-liquid extraction (LLE) was carried out using a mixture of methanol/chloroform (2/1 by volume; 600 μl). After mixing for 30 seconds with a vortex, chloroform (200 μl) and water (200 μl) were sequentially added, thoroughly stirring after each addition. The samples were centrifuged for 15 minutes at 3500 x g at room temperature. The organic (lower) phase was collected, dried under a nitrogen stream, and resuspended in 100 μl methanol/chloroform (9:1) for LC-MS/MS analysis. Analyses were conducted on a UPLC Acquity system coupled to a Synapt G2 QToF mass spectrometer (Waters). Samples were analyzed using a reversed-phase C18 T3 column (2.1 x 100 mm) kept at 55°C at a flow rate of 400 μl/min. The following gradient conditions were utilized: eluent A was 10 mM ammonium formate in 60:40 ACN/water, eluent B was 10 mM ammonium formate in 90 / 10 isopropyl alcohol / acetonitrile; after 1 minute at 30%, solvent B was brought to 35% in 3 minutes, then to 50% in 1 minute, and then to 100% in 13 minutes,

followed by a 1-minute 100% B isocratic step and reconditioning to 30% B. Total run time was 22 minutes. Injection volume was set at 3 ml. The capillary voltages were set at 3 kV and 2 kV for ESI+ and ESI-, respectively. The cone voltages were set at 30V for ESI+ and 35V for ESI-. The source temperature was 120°C. Desolvation gas and cone gas (N2) flow were 800 and 20 l/h, respectively. Desolvation temperature was 400°C. Data were acquired in MSe mode2 with MS/MS fragmentation performed in the trap region. Low-energy scans were acquired at fixed 4eV potential and high-energy scans were acquired with an energy ramp from 25 to 45 eV. Scan rate was set to 0.3 seconds per spectrum. Scan range was set to 50 to 1200 m/z. Leucine enkephalin (2 ng/ml) was infused as lock mass for spectra recalibration. Raw data from high resolution LC-MS/MS runs were subjected to principal component analysis (PCA) using MarkerLynx software (Waters). Metabolite accurate mass and retention time values were included in the multivariate analysis and assigned as X-variables. Orthogonal projection to latent structures discriminant analysis (OPLS-DA) and scatter plots were used to identify metabolites whose abundance was significantly altered between the experimental groups ($P < 0.01$ was set as minimum threshold for significance). To evaluate the over/under expression value, the peak area of each metabolite was normalized to the total metabolite intensity to generate a normalized peak area. Detected features were exported in a Microsoft Excel datasheet and subjected to two-tailed t-test for significance. Analytes were identified by interrogating the METLIN, HMDB, and LipidMaps databases. Tolerance on m/z values was set to 5 ppm. Identification was based on both accurate mass and calculated brute formula matching and, whenever possible, confirmed with tandem mass data.

Real-time PCR and microarray hybridization and data acquisition

Total RNA was extracted with Trizol (Invitrogen) according to the manufacturer’s instructions, purified using RNeasy MinElute Cleanup Kit (QIAGEN), and reverse-transcribed into cDNA using the iScript Reverse Transcription Supermix (Bio-Rad). Gene expression was measured by quantitative real-time PCR using 7900 HT Fast Real-Time PCR System (Applied Biosystems). Relative gene expression was determined using the ΔΔCT method. The list of primers is provided in **Supplementary Table 5**.

For microarray analysis, total RNA was purified using RNeasy MinElute Cleanup Kit (QIAGEN). RNA quality was analyzed by microfluidic gel electrophoresis on RNA 6000 Nano Chip using the Agilent 2100 Bioanalyzer (Agilent Technologies). RIN values ranged from 8.8 to 9.1, indicating high-quality total RNA. Cyanine-3 (Cy3)-labeled cRNA was prepared from 0.2 µg RNA using the One-Color Low Input Quick Amp Labeling Kit (p/n 5190-2331, Agilent Technologies) according to the manufacturer's instructions, followed by RNeasy column purification (QIAGEN). Dye incorporation and cRNA yield were analyzed with the NanoDrop ND-1000 Spectrophotometer (Nano-Drop Technologies). 1.65 µg Cy3-labeled cRNA (specific activity: 24.4 ±1.7 pmol Cy3 per µg cRNA) was fragmented at 60°C for 30 minutes in a reaction volume of 55 µl containing 1x Fragmentation Buffer and 2x GE (Gene Expression) Blocking Agent (Gene Expression Hybridization Kit, p/n 5188-5242, Agilent Technologies) following the manufacturer’s instructions. On completion of the fragmentation reaction, 55 µl 2x Hi-RPM Hybridization Buffer were added to the fragmentation mixture and hybridized to Agilent Mouse GE 4x44K V2 Microarray kit (G4846A, Agilent Technologies) for 17 hours at 65°C in a rotating hybridization oven. After hybridization, microarrays were washed for 1 minute at room temperature with GE Wash Buffer 1 and 1 minute at

37°C with GE Wash buffer 2 (Gene Expression Wash Buffer Kit, p/n 5188-5327 Agilent Technologies). Slides were scanned immediately after washing on the Agilent DNA Microarray Scanner (G2505C) using the AgilentHD_GX_1Color Profile (scan area: 61 x 21.6 mm; scan resolution: 5 µm, dye channel set to 100% Green PMT) of Agilent ScanControl software 8.1.3. The scanned images were analyzed with Feature Extraction Software 10.7.3.1 (Agilent Technologies) using default parameters (protocol GE1_107_Sep09). Expression data were analyzed using the limma package from R and false discovery rate (FDR) control for statistical assessment of the microarray data (corrected $P < 0.05$ were considered significant). Gene set enrichment analysis (GSEA) was performed to identify sets of related genes altered in each experimental group (http://www.broad.mit.edu/gsea). Heat maps were generated using MeV TM4 software V 4.9 (http://www.tm4.org). Microarray data are available at the Gene Expression Omnibus database under accession number GSE68441.

Mitochondrial membrane potential and complex activity analyses

Mitochondrial membrane potential was measured in isolated fibers from flexor digitorum brevis muscles. Mitochondrial membrane potential was measured by epifluorescence microscopy based on the accumulation of tetramethyl rhodamine methyl ester (TMRM) fluorescence. Briefly, flexor digitorum brevis myofibers were placed in 1 ml Tyrode's buffer and loaded with 2 nM TMRM (Molecular Probes) supplemented with 1µM cyclosporine H (a P-glycoprotein inhibitor) for 30 minutes at 37°C. Myofibers were then observed at Olympus IMT-2 inverted microscope equipped with a CellR imaging system. Sequential images of TMRM fluorescence were acquired every 60 secs with a 20X 0,5, UPLANSL N A objective (Olympus). At the times indicated by arrows, oligomycin (Olm, 5 µM) (Sigma) or the protonophore carbonyl cyanide *p*-trifluoromethoxyphenylhydrazone (FCCP, 4 µM) (Sigma) were added to the cell culture medium. TMRM staining was monitored in at least 10 fibers per group. Images were acquired, stored, and analysis of TMRM fluorescence over mitochondrial regions of interest was performed using ImageJ. For mitochondrial complex activity, total muscle lysates were extracted and the enzymatic activities of the respiratory chain complexes I-IV were assayed in duplicate or triplicate with a single-wavelength, temperature-controlled spectrophotometer at 37°C, as previously described [4]. The enzymatic activities for each mitochondrial enzyme was calculated as nmol min−1 mg−1 of protein and normalized to the activity of CS where indicated, which was used as a marker of the abundance of mitochondria.

Analysis of muscle force

To measure muscle force in living animals, the contractile performance of gastrocnemius muscle *in vivo* was measured as previously described [1]. Briefly, anesthetized mice were placed on a thermostatically controlled table, keeping the knee stationary, and the foot firmly fixed to a footplate, which was connected to the shaft of the motor of a muscle-lever system (305B, Aurora Scientific). Contraction was elicited by electrical stimulation of sciatic nerve. Teflon-coated seven-stranded steel wires (AS 632, Cooner Sales) were implanted with sutures on either side of the sciatic nerve proximal to the knee before its branching. At the distal ends of the two wires, the insulation was removed, and the proximal ends were connected to a stimulator (S88, Grass). To avoid recruitment of the dorsal flexor muscles, the common peroneal nerve was cut. For *in vitro* muscle mechanics, force measurements

performed *ex vivo* on isolated muscles are described elsewhere [1]. Briefly, isolated soleus muscles were mounted between a force transducer and a micro-manipulated shaft, and placed in an oxygenated organ bath and kept at 25°C. Optimal length was determined by stepwise lengthening the muscle while performing tetanic stimulation (80 HZ) until force production was maximal. Muscle force was normalized for cross sectional area, which was determined by measuring muscle mass and optimal length. The force-frequency relationship was determined by stimulating the muscle placed at optimal length and measuring the force at different frequencies.

Intraperitoneal glucose and insulin tolerance tests

Blood glucose was assayed with an Accu-Chek glucose monitor (Roche Diagnostics Corp.). Serum insulin levels were determined by ELISA using mouse insulin as a standard (Millipore). Glucose tolerance test was performed on 18 hr-fasted mice injected i.p. with D-glucose (2 g/kg body weight). Blood glucose levels were determined immediately before and 15, 30, 60, and 120 min after injection. Insulin tolerance test was performed on 4 hr-fasted mice upon intraperitoneal injection with human insulin (0.75 U/kg body weight, Humulin R, Eli Lilly). Blood glucose levels were determined immediately before and 15, 30, 60, and 120 min after injection.

Statistical analysis

All data are presented as mean ± standard error of the mean (SEM). Statistical differences of continuous data from two experimental groups were calculated using two-sample t-tests. Comparisons of data from more than two groups were performed using a one-way-ANOVA followed by a Fisher's least significant difference post hoc test. Statistical comparisons of lifespan curves were performed using the log-rank and Gehan-Breslow-Wilcoxon tests. Statistical significance threshold was set at $P < 0.05$, unless otherwise indicated.

Supplementary references

1 Blaauw B, Canato M, Agatea L, Toniolo L, Mammucari C, Masiero E, Abraham R, Sandri M, Schiaffino S, Reggiani C (2009) Inducible activation of Akt increases skeletal muscle mass and force without satellite cell activation. FASEB J 23: 3896-3905 Doi fj.09-131870 [pii]
10.1096/fj.09-131870

2 Kornberg A (1955) Lactic dehydrogenase of muscle. In: Colowick SP, Kaplan NO (eds) Methods in Enzymology. Academic Press, City

3 Mo K, Razak Z, Rao P, Yu Z, Adachi H, Katsuno M, Sobue G, Lieberman AP, Westwood JT, Monks DA (2010) Microarray analysis of gene expression by skeletal muscle of three mouse models of Kennedy disease/spinal bulbar muscular atrophy. PLoS One 5: e12922 Doi 10.1371/journal.pone.0012922

4 Spinazzi M, Casarin A, Pertegato V, Salviati L, Angelini C (2012) Assessment of mitochondrial respiratory chain enzymatic activities on tissues and cultured cells. Nat Protoc 7: 1235-1246 Doi nprot.2012.058 [pii]
10.1038/nprot.2012.058

5 Yu Z, Dadgar N, Albertelli M, Gruis K, Jordan C, Robins DM, Lieberman AP (2006) Androgen-dependent pathology demonstrates myopathic contribution to the Kennedy disease phenotype in a mouse knock-in model. J Clin Invest 116: 2663-2672 Doi 10.1172/JCI28773
